# Sex-specific longitudinal reversal of aging in old frail mice

**DOI:** 10.18632/aging.206304

**Published:** 2025-08-21

**Authors:** Cameron Kato, Jessica Zheng, Cindy Quang, Sophia Siopack, Joana Cruz, Zachery R. Robinson, Nicole Fong, Zhixin A. Zhang, Patrick Young, Michael J. Conboy, Irina M. Conboy

**Affiliations:** 1 Department of Bioengineering and QB3 Institute, University of California, Berkeley, Berkeley, CA 94720, USA; 2 Current address: College of Medicine, University of the Philippines Manila, Manila, NCR 1000, Philippines

**Keywords:** lifespan, healthspan, Alk5 inhibitor, oxytocin, sex-specific differences

## Abstract

Important studies report acute rejuvenation of mammalian cells and tissues by blood heterochronicity, old plasma dilution, defined factors, and partial reprogramming. And extension of rodent lifespan via single-prong methods was tried in recent years. Here, we examined whether simultaneous calibration of pathways that change with aging in opposite directions would be more effective in increasing healthspan and lifespan. Moreover, we started with the challenging age group - frail 25-months-old mice that are equivalent to ~75-year-old people. We used an Alk5 inhibitor (A5i) of the age-elevated, pro-fibrotic transforming growth factor-beta (TGF-β) pathway that regulates inflammatory factors, including IL-11, and oxytocin (OT) that is diminished with age and controls tissue homeostasis via G-protein-coupled receptor and ERK signaling. Treatment of old frail male mice with OT+A5i resulted in a remarkable 73% life extension from that time, and a 14% increase in the overall median lifespan. Further, these animals had significantly increased healthspan, with improved physical performance, endurance, short term memory, and resilience to mortality. Intriguingly, these benefits manifested only in the male and not in the female mice, yet OT+A5i had positive effects on fertility of middle-aged female mice. Mechanistically, the bio-orthogonal metabolic proteomics on the blood serum demonstrated that the acute, 7-day, treatment of the old mice with OT+A5i youthfully restored systemic signaling determinants and reduced protein noise in old mice of both sexes. However, after 4 months of OT+A5i, only old male, but not female, mice remained responsive, showing the youthful normalization of systemic proteome. These findings establish the significant health-span extension capacity of OT+A5i and emphasize the differences in aging and in response to longevity therapeutics between the sexes.

## INTRODUCTION

Extending lifespan and healthspan remains a central goal of biomedical research and has been tackled through numerous and diverse approaches. Transgenic models have provided insight into mechanisms of aging and rejuvenation, including Ames dwarf mice with the Prop1^df^ gene [1, 2], growth hormone receptor/binding protein (GHR/BP) knockout mice [3, 4], and p66^shc+/−^ knockout mice [5]. However, safe, scalable, and effective gene therapy remains clinically challenging and defined pharmacological interventions offer easier routes to clinical application.

Several pharmacological interventions have shown promise in extending lifespan, but each carries important limitations. One of the best known, rapamycin, targets the mTOR pathway and extends the lifespan of middle-age and old mice [6, 7]. However, its efficiency has varied between studies by different groups, and concerns over safety persist. Rapamycin associates with increased development of cancers, even with a transient administration [8], which may be linked to its clinical use as an immunosuppressant [9]. Adding a chemotherapy drug, trametinib, to rapamycin might mitigate the development of cancers and this drug mix was shown to extend mouse lifespan [10]; however, mTOR and mitogen-activated protein kinase, MEK, which these drugs inhibit, are needed for viability and vigor of many cells, including tissue stem cells and neurons [11]. Genetic knockout or antibody neutralization of IL-11 extend mouse life- and healthspans [12]. However, IL-11 is essential for ovarian health, and hematopoiesis [13, 14] and the levels of this interleukin increase in severe illnesses, but not in healthy human aging [15]. Another prominent direction in aging research is on senescent cells, which are targeted by a class of compounds known as senolytics, reviewed in [16]. These drugs compromise cell viability, their selectivity is imperfect, the long-term side effects of senolytics remain poorly understood, and senescent cells play a positive role in wound healing [17].

Yet another compelling avenue of longevity research centers on youthful modification of systemic environment, which is a determinant of tissue health, with age-imposed systemic changes promoting numerous pathologies [18, 19]. Research in this direction was pioneered by studies on heterochronic parabiosis and continued through heterochronic blood exchanges and in-vivo dilution of blood plasma; the latter established the capacity to acutely rejuvenate not only multiple organs in mice, but the cellular and humoral blood compartments in old people [20–22]. The dilution effects of therapeutic plasma exchange (TPE) are robust but last only transiently, and as this procedure involves processing the patient’s blood volume several times over in real time, which entails a variety of potential side effects [23–26]. Therefore, identifying the molecular determinants behind the benefits of TPE may be the key to developing safe and effective longevity therapeutics.

In pursuit of a systemic pathway modifier, we focused on a combination of OT (which naturally declines with age) and A5i, an inhibitor of ALK5 TGF-β receptor (targeting the age-associated physiologic increase in TGF-β signaling); in mice, this mix was shown to effectively rejuvenate muscle, liver, and brain after 7-day administration [27]. The TGF-β pathway becomes over-pronounced in the old, systemically and in tissues [28]. This pathway operates in a threshold fashion where healthy young levels are needed for homeostasis of cells and tissues, but the age-related increase promotes inflammation and interferes with regenerative capacity of tissue stem cells [29, 30]. OT, a nonapeptide best known for its role in child-bearing and social bonding [31], is critical for maintenance and repair of muscle, bone, etc. tissues and for healthy metabolism [32, 33]. OT acts on muscle and bone directly, through OT receptors expressed by these tissues, via downstream activation of MAPK/pERK [34].

Due to the complex and multifactorial nature of aging, we posited that any single factor might not suffice, at physiologic transient doses, for restoring health to old tissues, while chronic, supraphysiological doses might be potentially pathogenic. Thus, we combined inhibition of age-elevated TGF-β signaling, using A5i, with the administration of age-diminished OT and developed a precise dose of A5i, which when mixed with OT, allowed lowering the TGF-β antagonist by 10-fold, precluding overtly diminished signaling, yet broadened the transient rejuvenation effects [27].

Our results presented here establish that the longitudinal treatment of aged frail male mice with OT+A5i extended both lifespan and healthspan. Interestingly, while 7-day administration of OT+A5i youthfully normalized the systemic proteomes of both old males and old females, the female mice lost responsiveness to this therapeutic over time, based on the pattern of the systemic proteome, and had no benefits for life or health-spans. The causes of this sex-based differences remain unknown, albeit we note that treatment of female mice with OT+A5i at late middle-age rejuvenates fertility. The overall goal, which was achieved for male mice, was to avert morbidity and extend lifespan after the point of clear age-related decline, in a paradigm of true rejuvenation.

## RESULTS

### Longitudinal *in vivo* delivery of Alk5 inhibitor and oxytocin and effects on lifespan

To determine the longitudinal effects, 12 male control, 14 male OT+A5i, 13 female control, and 10 female OT+A5i animals were studied. The rationale for this combinatorial two drug treatment and the doses of the OT and A5i were chosen based on the dose-curve studies of the acute short-term rejuvenation [27]. The mice began the study at 25 months of age (see Materials and Methods for details) and were already frail; and they were randomly assigned to the experimental or control groups.

**Figure 1:**
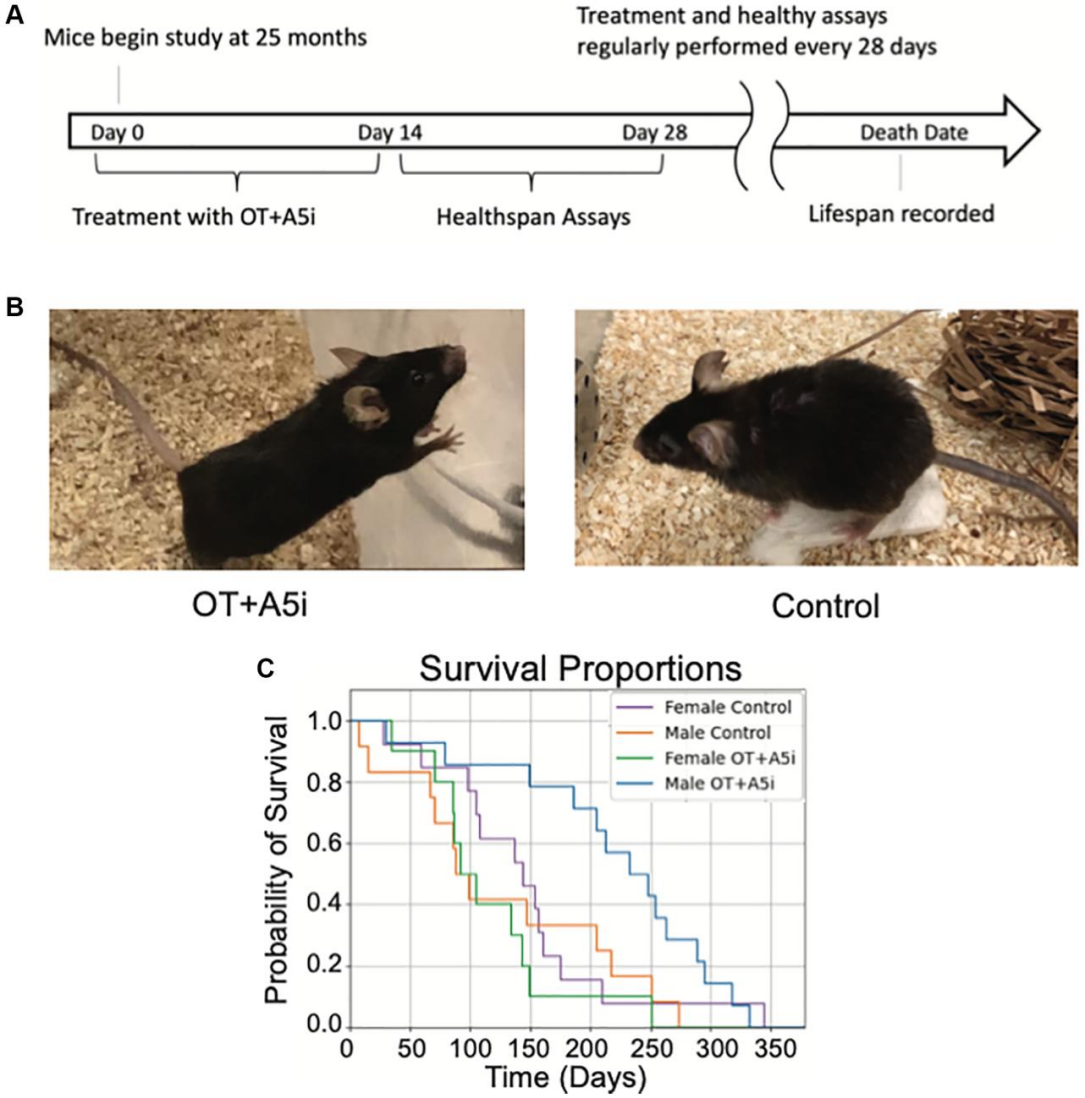
**OT+A5i extends the lifespan of old frail male, but not female mice.** (**A**) Outline of the longitudinal study. Old (24–26 mo) C57BL/6J mice were administered by subcutaneous injections with a mixture of OT and A5i (OT+A5i) or HBSS (control) for 2 weeks, followed by 2 weeks of health tests. These rounds were continued for the duration of the natural lifespan. (**B**) Photos of OT+A5i and control mice. (**C**) Survival curves of male and female mice that were administered with OT+A5i or control vehicle (Control). Male OT+A5i vs. Control (*p* = 0.0125). Control *n* = 12 and OT+A5i *n* = 14. Female OT+A5i vs. Control (*p* = 0.1904). Average survival time of female OT+A5i is 115.3 days. Average survival time of female controls is 144.7 days. Control *n* = 13 and OT+A5i *n* = 10.

Mice were injected subcutaneously with OT (1 μg/g-day) and A5i (0.02 nmol/g-day) or vehicle control (HBSS) cyclically every Monday, Wednesday and Friday for two weeks, followed by a two-week rest period before the next round of injections (Figure 1A). The health and frailty studies were conducted on these animals until their natural endpoint.

Upon visual observation, male mice treated with OT+A5i looked notably healthier and less frail as compared to old controls (Figure 1B) and one of the most robust effects of OT+Al5i was to significantly expand the lifespan (Figure 1C**)**. Namely, there was a 14% increase in median total lifespan (counting from birth) in the OT+A5i male mice. But note as these mice were already old when the treatment started, counting from the time of first administration there was a very impressive 73.73% increase in additional life: OT+A5i male mice lived on average 221.1 additional days while male controls lived only 127.3 additional days from the start of the study (Figure 1C). Measuring median additional life, OT+A5i male mice lived 240.5 median days post-injection, 157.22% longer than the control male mice, which lived only 93.5 median days post-injection. Moreover, the death hazard ratio (likelihood of death) was almost three times higher (2.868) for the control male mice compared to the OT+A5i male group based on the Cox proportional hazards model.

The full lifespan data is shown in (Supplementary Figure 1) where our results are plotted against the published lifespan curve of the Mouse Phenome Database [35], rigorously confirming the significant improvement in lifespan of the old frail male mice by OT+A5i.

Surprisingly, and unfortunately, there was no improvement in the lifespan of the female mice (Figure 1C and Supplementary Figure 1), demonstrating clear sex-related differences in responsiveness to OT+A5i. The hazard ratio of Control vs. OT+A5i female mice is 0.5663.

### Longitudinal effects of OT and A5i on healthspan

**Figure 2:**
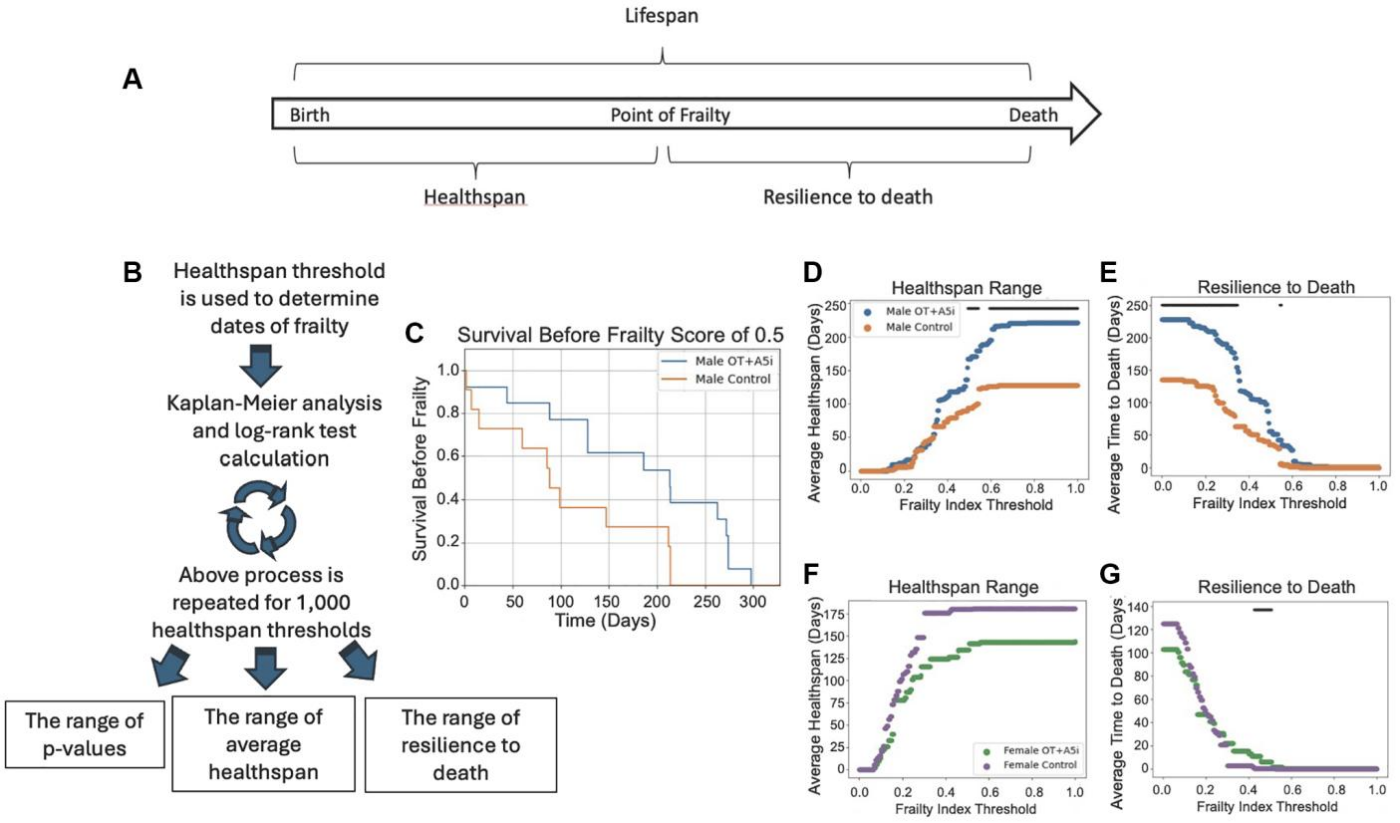
**A5i+OT extends the healthspan of old frail male mice.** (**A**) Overview of lifespan vs. healthspan and related terms. (**B**) Outline of the novel statistical model created to analyze healthspan. Healthspan thresholds are used to determine the dates of incurred frailty and log-rank test analysis is performed. This is repeated 1,000 times for a range of healthspans and the average healthspans are plotted as well as the resilience to death. (**C**) Representative Kaplan-Meier plot demonstrating the healthspan of mice before they reach a frailty of 0.5 (*p* = 0.0230). (**D**) The average healthspans of OT+A5i and control mice across the range of frailty thresholds from 0 to 1. The black bar represents thresholds at which there is a statistically significant difference as calculated by log-rank analysis. The maximum significant frailty index value threshold = 1.0 and the minimum threshold = 0.4925. (**E**) The average time to death after the frailty threshold is reached, which we denote as “resilience to death.” Male mice: control *n* = 12 and OT+A5i *n* = 14. The maximum significant frailty index value threshold = 0.5495 and the minimum significant frailty index value = 0.0. (**F**) The average healthspans of OT+A5i and control female mice across the range of frailty thresholds from 0 to 1. The black bar (or lack thereof) represents thresholds at which there is a statistically significant difference as calculated by log-rank analysis. There were no thresholds at which a statistically significant difference was observed. (**G**) The average time to death after the frailty threshold was reached for female mice: “resilience to death”. Female mice: control *n* = 13 and OT+A5i *n* = 10. The maximum significant frailty index value threshold = 0.5075 and the minimum significant frailty index value = 0.4254.

In addition to lifespan, we studied the healthspan of the aged frail mice that were longitudinally administered with OT+A5i or HBSS control. During the 2-week rest intervals between the OT+A5i treatments, health tests were performed including treadmill, novel object recognition, 4-limb hanging tests, and multiplex frailty scoring (Figure 2A).

Further, we quantified the loss of health below several distinct thresholds, using a Kaplan-Meier plot with the analyses of the log-rank test: the longitudinal statistics that inherently accounts for dropout of data-points, such as those due to mouse morbidity (Figure 2B).

To increase the clinical relevance and the rigor, instead of evaluating healthspan in buckets of age, we tested different health decline thresholds to determine what phase of health, if any, was improved and when. For each of these thresholds, we used multi-parametric frailty scoring (Figure 2C–2E), as well as treadmill, 4-limb hanging, and cognitive novel object recognition tests (next result section) to individually and collectively inform on multiple aspects of health. The frailty assessment combines 31 different metrics to produce one frailty index (FI) [36]. This includes assessments of the vision, gait, body condition, coat condition, grip strength, and more (see Materials and Methods).

Data analysis with the healthspan statistical model, which accounts for the differential mortality within the cohorts, demonstrated that longitudinal administration of OT+ A5i improved survival scores of old male mice at 0.5 frailty index threshold (Figure 2C**)** and increased the average healthspan of old frail male mice, with trends at a variety of health thresholds, but most robustly with highest statistical significance at higher frailty thresholds (Figure 2D). This suggests that with more rounds of OT+A5i, the positive effects on the healthspan became stronger. Namely, as compared to vehicle control, the OT+A5i treated male mice were significantly healthier at a point of frailty of 0.5, and the difference between the healthspan of these mice vs. the control vehicle mice increased further at the higher frailty thresholds. These data further substantiated that with extended time on OT+A5i, the healthspan of the male mice continued to improve, and the animals remained healthier for longer. Furthermore, the male mice treated with OT+A5i were much more resilient to death: even after reaching a wide range of frailty thresholds, they survived for significantly longer compared to control male mice (Figure 2E).

No improvement in the health span or in the resilience to death was observed for female mice that were treated with OT+A5i, echoing the lack of increase in their lifespan (Figure 2F, 2G). In female mice, there was even a non-statistical trend for diminished healthspan range with OT+A5i, as compared to vehicle control. This was despite the fact that the initial frailty of the females was lower than that of males for both control and OT+A5i groups, and that there was no statistical difference in initial animal frailty between the control vehicle vs. OT+A5i (Supplementary Figure 2 and Supplementary Data 1).

### Longitudinal effects of A5i+OT on physical performance, endurance, balance/agility and short-term memory

Next, we decided to examine in more detail the functional performance of the mice that started the study as old frail animals on the treadmill (endurance), in a 4-limb hanging test (balance, agility, memory), and novel object recognition test (short term memory).

In the treadmill test, mice are placed on a treadmill which increases pace every 10 minutes [37], beginning the test at a slower pace of 8 m/min and increasing the speed by 1 m/min every 10 minutes, until 60 minutes, after which it was increased every 5 minutes.

**Figure 3:**
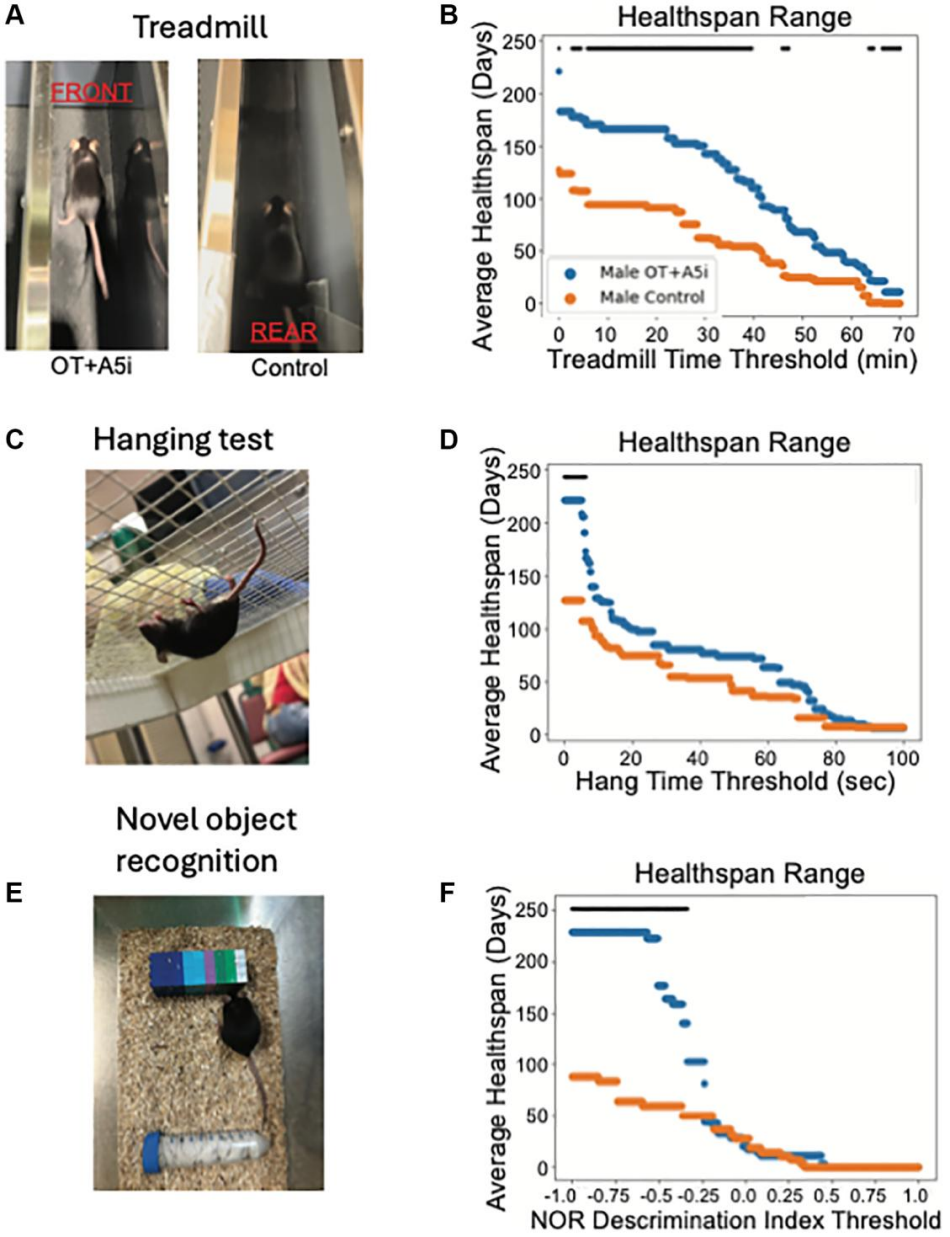
**OT+A5i enhances endurance, balance/agility/grip strength and memory of old frail males.** (**A**) Mice were placed on a treadmill at a rate of 8 m/min and the treadmill speed was increased at a rate of 1 m/min every 10 min for a total of 60 min and then was increased at 1 m/min every 5 min until mice shifted toward the rear of the treadmill. (**B**) The average healthspans of OT+A5i and control mice across the range of treadmill running time thresholds from 0 to 70 minutes. Control *n* = 12 and OT+A5i *n* = 14. (**C**) Mice were placed on a grid and inverted 25 cm over soft bedding. After three trials their average hanging time was calculated. (**D**) The average healthspans of OT+A5i and control mice across the range of hang test time thresholds from 0 to 100 seconds. The maximum significant threshold = 6.006 seconds and the minimum significant threshold = 0.0 seconds. Control *n* = 12 and OT+A5i *n* = 14. (**E**) In NOR test, mice were first exposed to two identical objects for 10 minutes, followed by a 2-hour rest and exposure to the habituated object and a novel object for another 10 minutes. The discrimination index describes their interaction preferences, with 1 indicating a preference for the novel object and 0 for the habituated object. (**F**) The average healthspans of OT+A5i and control mice across the range of NOR discrimination index thresholds from 1 to −1. Control and OT+A5i *n* = 6 each. The maximum significant threshold = −0.341 and the minimum significant frailty index value = −1.0. The black bar is the threshold at which there is a statistically significant difference by log-rank analysis, in (**B**, **D** and **F**).

Using the health thresholds statistics, we found that OT+A5i increased the treadmill-based healthspan of male mice, as compared to the negative control vehicle (Figure 3A, 3B). Supplementary Figure 3A–3C confirms this improvement through several Kaplan-Meier healthspan thresholds of the old male mice at different running times on the treadmill.

To assess the grip strength, coordination, balance and agility of the mice, we used the four-limb hanging test [20, 38]. In the test, mice are placed on a mesh screen and inverted over soft bedding; the time until the mouse drops is recorded over three trials, and the average is taken. While there was a portion of the 4-limb test based healthspan with a statistically significant difference between male OT+A5i vs. control male mice, this change was not as strong as seen in the treadmill test (Figure 3C, 3D).

To evaluate the cognition and short-term memory of the mice, we analyzed their healthspan in the novel object recognition test (NOR) [39, 40]. In NOR, mice are first exposed to two identical objects to habituate them for 10 minutes. Following this, they are given a two-hour resting period before being exposed to the habituated object and a novel object. The discrimination index describes the preference for the novel object, with higher cognitive capacity and short-term memory associated with a higher index score. Consistently with the positive effects of the short-term OT+A5i [27], the longitudinal treatment significantly improved the cognition based healthspan of the aged male mice (Figure 3E, 3F). In contrast to males, no improvements in the studied parameters of health were found in the old frail female mice, treated with OT+A5i (Supplementary Figure 3D–3F, and Supplementary Data 1).

Interestingly however, when tested in middle-aged females, OT+A5i treatment improved a key age-diminished metric of fertility, resulting in higher numbers of weaned pups in 8–12 months old female mice (Supplementary Table 1).

### Acutely, OT+A5i youthfully calibrates *de novo* systemic proteome in old mice of both sexes

We examined the acute influences of OT+A5i on the blood serum proteome, using high-resolution bio-orthogonal metabolic proteomics to identify the specific changes in *de-novo* synthesized, systemically present proteins [41, 42]. In this system, a methionine analog is injected into mice and is ubiquitously incorporated into the newly synthesized proteins. This methionine analog contains an azide group that can then be detected by click chemistry.

**Figure 4:**
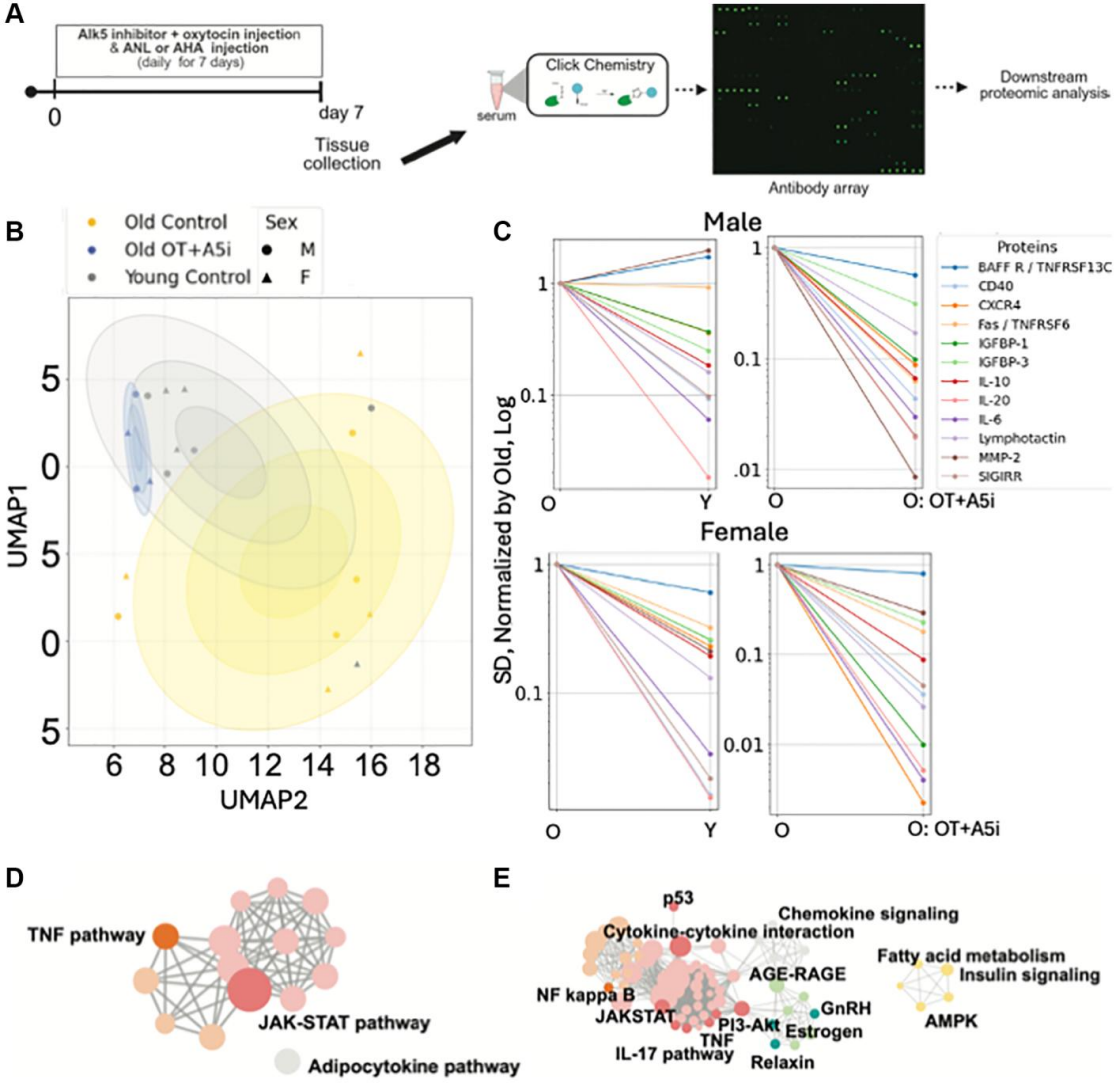
**Acute effects of OT+A5i on *de novo* systemic proteomes of males and females.** (**A**) Schematics of the experimental approach. (**B**) Uniform Manifold Approximation and Projection (UMAP) plots of the profiles of *de-novo* synthesized systemically present proteins of young control, old control, and old OT+A5i mice, males and females. AHA and ANL data are merged. (**C**) Sex-annotated comparison of the mean SDs of the Levene’s test passed proteins (*p* < 0.05) with lower noise in young control vehicle mice compared to old control vehicle mice and in old mice administered with OT+A5i vs. vehicle control. In the shown line graphs, SDs of all groups are normalized by the SDs of the old vehicle control and presented on a log scale. (**D**) cirFunMap visualization of KEGG enriched pathways of *de-novo* synthesized systemic proteomes after OT+A5i treatment. (**E**) cirFunMap visualization of KEGG enriched pathways of the *de-novo* synthesized proteins that are noisier in the old as compared to young mice and become youthfully normalized by OT+A5i in old males and females. *n* = 4 each young and old MetRS^L274G^ vehicle control, *n* = 4 each young and old C57.B6 vehicle control, *n* = 4 old OT+A5i C57.B6.

The mice were administered with OT+A5i or vehicle control for 7 days by daily subcutaneous injections. At the same time, azido-nor-leucin (ANL) was injected into the old transgenic mice, in which mutant methionine t-RNA synthetase (MetRS^L274G^) bio-orthogonally integrates ANL instead of methionine [41, 42]. In an additional setting, C57BL/6 mice were administered with azido-homo-alanine (AHA), which is integrated into *de-novo* proteins by the wild-type methionine t-RNA synthetase. This experimental design is illustrated in Figure 4A.

Blood serum of these animals was assayed via bio-orthogonal non-canonical amino acid proteomics (BONCAT), using RayBiotech BONCAT antibody arrays, as previously published [41].

The Uniform Manifold Approximation and Projection (UMAP) identified the different profiles of *de-novo* proteomes in the OT+A5i versus the vehicle control mice, males and females (Figure 4B). The *de-novo* synthesized serum proteins of the old, OT+A5i treated, mice clustered around the young vehicle cohort and differed from the old vehicle cohort, suggesting a youthful restoration of systemic proteome by OT+A5i, in both sexes.

Next, we examined the effects of OT+A5i on biological noise, which is a key biomarker of aging [21, 43]. We identified the Levene’s test passed *de-novo* synthesized proteins that vary in the standard deviations (SD) between the young control, the old control, and the old OT+A5i cohorts, as previously published [21]. To improve the rigor, we additionally applied the Benjamini-Hochberg false discovery rate analysis, to prevent potential over-detection of the differences in SDs [44]. We found several proteins (BAFF R, CD40, CXCR4, Fas, IGFBP-1, IGFBP-3, IL-10, IL-20, IL-6, Lymphotactin, MMP-2, SIGIRR) with increased noise (statistically higher SD values) in the old vs. the young control groups, albeit there were sex-specific differences in the young-to-old changes between the sexes; importantly, the noise of these proteins was diminished by OT+A5i in both old males and old females (Figure 4C).

Based on Kyoto Encyclopedia of Genes and Genomes (KEGG) bioinformatics, the *de-novo* synthesized youthfully normalized by OT+A5i signaling determinants of TNF and JAK-STAT pathways were the top enriched terms (Figure 4D). With respect to the age-altered and OT+A5i normalized systemic determinants of protein noise, NF kappa B, JAK-STAT, PI3-Akt, TNF, AGE-RAGE, and AMPK, were highly enriched (Figure 4E). Supplementary Figure 4 shows additional analyses of this comparative *de-novo* proteomics via the sex-annotated Heatmap on DEPs and the merged between males and females protein noise curves. The full BONCAT data are provided in the Supplementary Data 1.

Overall, these results establish that even though female mice did not show life- and health-span benefits of OT+A5i, in the short term this pharmacology promotes younger states of systemic proteome in both sexes.

### After 4 months of OT+A5i treatment, systemic proteome is youthfully calibrated in male but not in female mice

**Figure 5:**
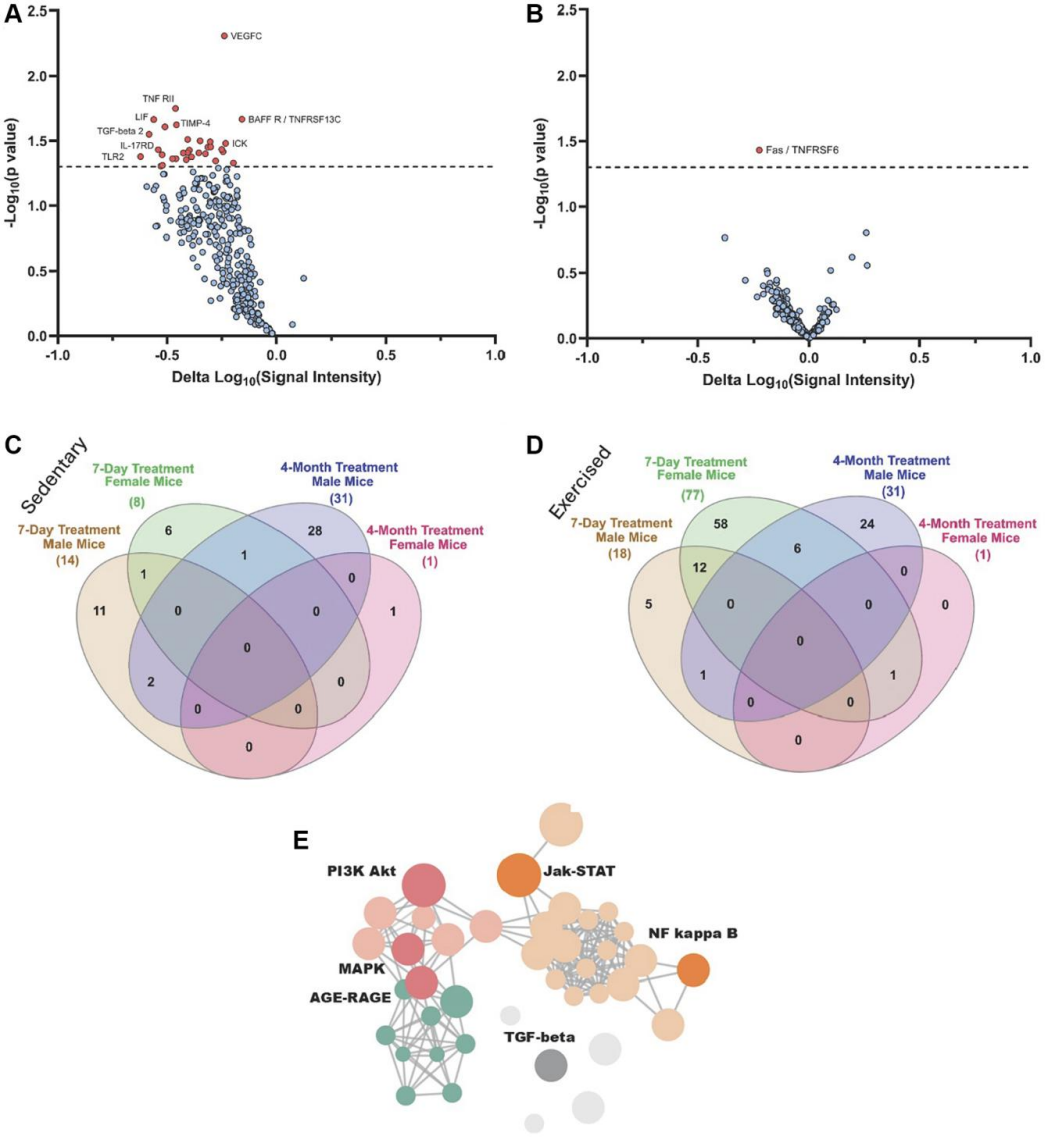
**Proteomic changes in male and female mice after 4-months of OT+A5i vs. vehicle.** (**A**) Volcano plot showing DEPs in male mice, based on antibody arrays data. (**B**) Volcano plot showing DEPs in female mice, based on antibody arrays data. (**C**) Venn diagram on the overlap of DEPs (*p* < 0.05 OT+A5i vs. vehicle control) between sedentary 7-day treated males and females, and 4-month treated males, and females. (**D**) Venn diagram on the overlap of DEPs (*p* < 0.05 OT+A5i vs. vehicle control) between exercised 7-day treated males and females, and the same as in C 4-month treated males, and females. (**E**) Enrichment analysis for differentially expressed proteins in the male mice, using KOBAS with KEGG pathway analysis. 7-days *n* = 8 control vehicle (4 males, 4 females), *n* = 8 OT+A5i (4 males, 4 females). 4-months: *n* = 4 all cohorts, except, *n* = 3 OT+A5i males.

Next, we studied the serum proteomes of the mice after 4 months of OT+A5i treatment versus vehicle control (HBSS). The results of the RayBiotech arrays demonstrated that in the old male mice, which were administered with OT+A5i, numerous pro-inflammatory proteins were attenuated (Figure 5A). Interestingly, and in agreement with the lack of life- and health-span extension, OT+A5i failed to modulate the systemic proteome in the old female mice, at 4-months of administration (Figure 5B).

To better understand the mechanisms driving the differences between the acute effects of OT+A5i and the long-term responsiveness to this pharmacology, we compared the 7-day and 4-months proteomic datasets via Venn diagrams. Because the animals in our longitudinal study underwent regular treadmill exercises, we compared the proteomes of the 4-months treated mice to that of not only sedentary, but also treadmill exercised mice of the 7-day cohort. The systemic differentially expressed proteins, DEPs (*p* < 0.05 sex-matched OT+A5i vs. control vehicle), were compared between the total proteomes at 4-months of the treatments and the *de-novo* synthesized after 7 days of injections (Figure 5C, 5D). As compared to sedentary animals, the number of *de-novo* synthesized systemic DEPs was higher after exercise, particularly, for the old female mice, after the 7-day treatment; for the old males some of these DEPs were also present at 4-months; however, almost none were shared between the 4-months old female group and other cohorts (Figure 5C, 5D). Supplementary Figure 5 shows Volcano plots for the sedentary and exercised male and female mice, as compared to their sex-matched vehicle treated controls. The full list of proteins is provided in the Supplementary Data 1.

To gain further insight into the involved pathways, enrichment analysis was performed using the Kyoto Encyclopedia of Genes and Genomes (KEGG) and visualized through the KOBAS-i web platform. Key signaling pathways, which are important for cell and tissue homeostasis and associate with age-related pathologies when dysregulated, were found to be youthfully normalized in the old male mice by the long-term *in vivo* treatment with OT+A5i, such as Jak-STAT, MAPK, and AGE-RAGE networks (Figure 5E).

Thus, old female mice become unresponsive to the positive effects of this OT+A5i on the normalization of systemic proteome by 4 months of *in vivo* administration, while old male mice continuedly have beneficial effects of this pharmacology. These patterns of systemic proteomes correlate well with the sex-differences of the positive effects of OT+A5i on life- and healthspans.

## DISCUSSION

This work establishes that continuous re-calibration of two signaling pathways, which change with aging in opposite ways, is sufficient for robust rejuvenation of physical and cognitive capacities of already old and frail male animals, and moreover prolongs not only their lifespan, but increases their healthspan and resistance to morbidity.

One of the most interesting findings of this study is the clear sex-specific difference in the capacity of OT+A5i to expand the life- and health-spans. Female mice are generally understudied in the work on acute tissue rejuvenation by blood heterochronicity [18]. Yet sex-specific differences have been repeatedly found in lifespan and healthspan studies (metformin [45], rapamycin [8], acarbose [46], nordihydroguaiaretic acid [46, 47], Aspirin [47], 17-α estradiol [48]). The data points toward interesting sex-specific mechanisms of responses to longevity therapeutics, warranting further research.

Positive effects of OT+A5i on fertility of middle-aged females indicates that starting treatment earlier in life could be more effective for their healthspan extension. The mechanisms, by which OT+A5i therapeutic improves fertility would be interesting to establish, for example, performing analysis of the levels and the composition of fertility-related hormones. In humans, menopause leads to a sharp decline in estrogen and progesterone, however, female mice do not experience menopause in the traditional sense and instead reach a less severe state of reproductive senescence [49]. Interestingly, estrogen and oxytocin have positive feed-backs for each other and influence other reproductive hormones, e.g., oxytocin regulates activity of gonadotropin-releasing hormone (GnRH) neurons, which control luteinizing hormone and follicle stimulating hormone, leading to estrogen production in the ovaries [50]. Indeed, based on our data, estrogen was a participant in the protein networks that are modulated by OT+A5i at 7 days of administration. On the opposite end of this spectrum, TGF-beta promotes ovarian degenerative process, known as follicular atresia [51]. Thus, OT+A5i may improve fertility of older female animals through multiple mechanisms.

The hormonal landscape is linked to metabolism and interestingly, AgRP, which is reduced by OT+A5i, regulates lifespan, such that attenuation of AgRP promotes longer lifespan through changes in food consumption [52]. Moreover, at young healthy levels, AgRP neurons mediate activity-dependent oxytocin connectivity [53].

When considering the availability and safety for human use, it is notable that oxytocin is approved by the FDA, and it was in clinical practice for many decades [54]. A5i drugs, like Vactosertib [55] are undergoing clinical trials for treating cancer and myelodysplastic syndromes, and no major adverse side-effects were reported so far [56]. Thus, while being effective for multi-parametric rejuvenation, on par with non-clinically feasible heterochronic parabiosis, OT+A5i holds a promise of rapid medical translation and use as a health-expansion therapy.

Compared to other established lifespan-extending interventions, OT+A5i demonstrates unique outcomes, such as significantly (over 70%) increased life expectancy from the start of this therapy in old and frail male mice, and a robust decrease in mortality risk. In comparison, a meta-analysis of 29 rapamycin studies revealed that when measured from the start of the therapy female mice had a 15.1% increase in survivorship with a hazard ratio of 0.41, while male mice had only a 9.4% extension [57] and hazard ratio of 0.63. Metformin, another proposed lifespan-extending treatment, has shown variable results. In a study on 129/Sv mice, males had an increase in mean and median lifespan (measured from birth) by 20% and 19.3%, respectively with a hazard of 0.2, while females experienced a *decrease* in mean and median lifespan by 9.1% and 13.8%, respectively [45]. Yet, a study on Fischer 344 rats contradicted metformin’s efficacy as a life-extending therapeutic reporting the hazard ratio of 0.983 and no difference between the lifespans of control and treated groups [58]. According to a review of 53 studies, metformin’s potential as a lifespan therapeutic remains controversial, with ongoing debate over its aging-relevant mechanisms of action [59]. Therefore, OT+A5i is well-positioned as a promising, clinically feasible intervention for extending male life- and healthspan through systemic calibration of key signaling determinants, distinguishing it from other approaches.

In addition to the findings on rejuvenation via OT+A5i, our novel statistical approach provides a methodology to evaluate the entire range of healthspan. Previous studies on health of old, young, and middle-aged mice [8, 60, 61] did not account for the morbidity that invariably occurs in longitudinal studies. Since the least healthy, frailest animals typically die first, the health data becomes skewed. If in one cohort, all mice were to quickly lose health and die except for a most resilient mouse, one wouldn’t say that the average healthspan of that cohort is equivalent to a cohort with all healthy mice. Using our approach with log-rank analysis significantly improves the accuracy of the statistics on healthspan in experiments with morbidity-related dropout.

With respect to the mechanisms of the extension of life- and health-spans by OT+A5i, and only in male mice, our proteomics identified key sex-specific differences. Namely, in the old male mice, there were numerous DEPs, resulting in a youthful calibration JAK-STAT, both at 7 days and at 4 months of OT+A5i treatment. In old females, the changes in systemic proteome by OT+A5i were detectable at 7-days, increasing in the exercised group, which is consistent with the common knowledge that exercise promotes healthspan, however, no effects were seen at 4-months. The reasons for the sex-specific differences remain unknown and warrant future research. In the exercised old males and females, OT+A5i, interestingly, promoted protein synthesis of the regulators of angiogenesis, adaptive immunity, and homeostasis, similarly to the positive effects of old plasma dilution [20–22].

Regarding the beneficial effects of OT+A5i on the healthy longevity of old male mice, TNF and JAK-STAT pathways crosstalk with TGF-β, MAPK and AKT/mTOR through IL-1, IL-3, IL-15 [62–64], which were some of the DEPs identified in this study. Thus, the network of major aging-dysregulated signaling pathways becomes systemically recalibrated to a younger state. Reduction of inflammaging by OT+A5i is expected, as TNF pathway activates this process and also increases apoptosis and senescence, promoting muscle loss, platelet hyperreactivity and thrombosis [65–67]. Similarly, TGF-β and JAK-STAT are involved in chronic inflammation, senescence and age-associated diseases, such as cancer, tendinopathies, atopic dermatitis, and immunodeficiencies [68, 69]. Downregulation of TNF and JAK-STAT results in clinical benefits, including reduced inflammation and cellular senescence, increase in healthy tissue homeostasis, and improved outcomes for myopathies, rheumatoid arthritis, liver diseases, and cancer [69–71].

Further, TGF-beta is a key player in the activation and proliferation of Col1A2 expressing fibroblasts and the production of collagen [72, 73]. Aberrant signaling in the TGF-β pathway has been linked to several connective tissue disorders [74], and severe fibrotic pathologies of the lungs, kidneys and liver [75, 76]. Accordingly, inhibition of TGF-β signaling with A5i effectively blocks the progression of pulmonary fibrosis by reducing collagen levels [76]. In liver, A5i ameliorates overproduction of collagen and TIMP-1, attenuating fibrosis, etc., pathological changes in extra-cellular matrix [77]. Thus, while the long-term effects of A5i on collagen remodeling remain unknown and warrant further investigation, short-term treatment has shown promise in managing diseases of connective tissues. Interestingly, blocking IL-11 failed to attenuate the TGF-β-caused fibrosis [78]. Therefore, A5i, which breaks the auto-induction of TGF-β1 expression, might more optimally ameliorate certain age-related pathological hallmarks.

In summary, this study establishes OT+A5i as a promising, clinically viable strategy for extending lifespan and healthspan in aged, frail male mammals. Importantly, the favorable safety profiles of both components emphasize the translational feasibility for human use. The findings reveal intriguing sex-specific differences that warrant further mechanistic exploration and underscore the potential for more tailored therapeutic approaches in longevity medicine.

## MATERIALS AND METHODS

### Animals

All *in vivo* experiments and procedures were performed in accordance with the policies set by the Office of Laboratory Animal Care and under the approved protocol. Aged C57BL/6J mice (25 months old) were purchased from the National Institute of Aging.

### Breeding

Female mice were injected with either HBSS or OT+A5i every day for 7 days. Female mice were bred with young (3–7 mo) male mice at 8, 10 and 12 months of age.

### Oxytocin (OT)

Was purchased from Bachem (H-2510) and a 30mM stock prepared in sterile water.

### Alk5 inhibitor (A5i)

TGF-β1 Type I Receptor Kinase Alk5 inhibitor 2-(3-(6-Methylpyridin-2-yl)- 407 1H-pyrazol-4-yl)-1,5-naphthyridine, was purchased from Enzo Life Sciences, and a 25 mM concentrated stock dissolved in DMSO.

### Subcutaneous injections

OT and A5i or control vehicle (HBSS) were injected subcutaneously to old male C57BL/6J mice (25 months old), on Monday, Wednesday and Friday for two weeks before two weeks of health tests. This continued in a cycle every four weeks until the death of the mice.

### Azido-nor-leucine treatment

Mice were injected intraperitoneally with azidonorleucine (ANL) (Jena Biosciences CLK-AA009, 0.02 mmol/kg) daily for 7 days.

### 31-Item clinical frailty index test

The frailty index test was conducted as previously published [36]. 31 different metrics of health and frailty including items such as alopecia, tumors, and gait were all individually assessed and averaged together to make a composite index. This overall describes the frailty of the mice, which typically increases with age.

### Treadmill test

The treadmill test was conducted as previously published, with some modifications for the aged state of the mice [37]. The test consists of two days of habituation followed by a day of testing, which is described below.

#### Acclimation and training

All mice were acclimated to the treadmill (Columbus Instruments, Exer-6M Open Treadmill) 2 days prior to the exercise test session, as follows. Food was removed 2 hours before exercise. Mice were familiarized with the sounds and experiences of the moving treadmill for 2 sessions (1 session per day). Mice were put on the stationary treadmill for 30 seconds to explore the environment. Acclimation began at a low speed of 5 to 8 meters per minute (m/min) for a total 10 minutes on day 1 and increased to 5 to 10 (m/min) for a total 10 min on day 2. Following training, mice were allowed to rest for 1 day in their home cage before the test to exhaustion. If an animal did not adapt to the familiarization protocol, it was removed from the experiment and not subjected to the exhaustion test until the next round of tests in 4 weeks.

#### Exhaustion test

Food was removed 2 hours before exercise. The treadmill began at a rate of 8 m/min and the treadmill speed increased at a rate of 1 m/min every 10 min for a total of 60 min and then increased at a rate of 1 m/min every 5 min until mice were exhausted. Exhaustion was determined by refusal of mice to remain on the treadmill for at least 10 seconds. During the exhaustion test, mice were removed from the treadmill once considered to be exhausted, this time was recorded, and they were returned to the home cage. Once the time reached 90 minutes, mice were allowed to continue to run at speed 20 until exhausted.

The treadmill broke down in January of 2021, during which time some mice were unable to be tested until the unit was repaired by the following cycle in February of 2021.

### Four-limb hanging test

The four-limb hanging test was conducted as previously published, with some modifications for the aged mice [20, 38]. Mice are placed on a grid with the following dimensions: 1 cm mesh, 1 mm wire screen. Position the grid 25 cm above soft bedding to prevent mice from harming themselves upon falling, but also to discourage mice from intentionally jumping off the grid. The mouse will be placed on the grid so that it grasps it with its four paws. The grid will be inverted so that the mouse is hanging and following this, the time will be recorded. The test session ends for mice that are able to hang for a duration of 300 sec. Mice are given 3 attempts with at least a 5 min rest between trials and the average of the trials is calculated. If the mouse slips and fails to hang on for at least 10 seconds, it will be given an additional attempt, but not more than 2 additional attempts total.

### Novel object recognition test

The novel object recognition test was performed as previously published [39, 40]. Two identical objects will be placed in the arena, and the mouse will be allowed to explore for 10 minutes. Resting: The mouse will then be removed from the arena for 2 hours. At this point, the habituated objects will be removed and replaced with two new objects: the habituated object and a novel object. The familiar or novel object/texture should be randomly selected. Testing: The mouse will then be allowed to interact with these new objects for a final 10 minutes. The objects are sanitizable small objects: a plastic cylinder or a rectangular block made of different colored building bricks. Every object and/or texture column will be sanitized (sprayed with NPD (Non-Phenolic Disinfectant), rinsed and dried), prior to coming in contact with each set of mice from a cage.

### Serum collection

Blood was collected every month and at the end of the study. For the monthly blood collection, chin bleed method was performed. Briefly, mice were anesthetized using 2% isoflurane until unconscious. Blood (at most 150 uL) was collected by puncturing the chin with a 23G needle. No more than 10% of the animal’s total blood volume was removed per collection, as previously published [79]. For the terminal procedure, blood was collected through cardiac puncture using a 1-mL syringe with a 25G needle. Serum was isolated via centrifugation at 2,000× g for 10 minutes and stored at −80°C until used.

### Antibody arrays

Levels of 308 proteins (L-308 glass array, RayBiotech, Norcross, GA, USA) were measured in serum following manufacturer’s protocol. For BONCAT array, serum samples were clicked using Click-iT^®^ Protein Reaction Buffer Kit (Thermo Fisher Scientific C10276). Afterwards, clicked samples were dialyzed in 0.2% Triton X-100 in PBS. Alongside, the array membranes were incubated with blocking buffer overnight at 4°C. After blocking, the array membranes were incubated overnight at 4°C with 400 μg of clicked-dialyzed serum. After washing, the arrays were incubated with Streptavidin-conjugated Cy3 fluorescence dye overnight at 4°C. The array slides were washed, dried, and scanned using GenePix Pro 6.1 software (Molecular Devices, San Jose, CA, USA). Fluorescence intensities were obtained for each array. Array normalization was done by the built-in positive and negative controls.

### Uniform manifold approximation and projection (UMAP)

UMAP was conducted as previously published [80]. Confidence ellipses were created for visual clarity of groupings and represent 0.5, 1 and 1.5 standard deviations based on each cohort of mice’s coordinate values along PCA1 and PCA2.

### Noise analysis

We detected proteins with elevated noise as previously published [21]. We used Levene’s test to detect proteins that had significantly different levels in their standard deviation values and we applied the Benjamini-Hochberg procedure. We compared the values of the control old to the old OT+A5i, with both sexes combined and all treated with AHA. From this, we identified 12 proteins with decreased noise after OT+A5i treatment. The normalized SDs of these proteins were then plotted.

### Healthspan statistical analysis

The statistical analysis we used was a novel method to evaluate overall healthspan at a range of frailty points. For every metric of health, we determined the range of all points of frailty and proceeded to calculate at which point in time each animal passed these points of frailty. Within the range of points, 1000 equidistant different points were selected, and each was used to calculate a log-rank test statistic and the average time from the beginning of the study (25 months for mice) until the time point is graphed as the average healthspan. The logrank test is specifically built for longitudinal studies and inherently accounts for dropout of data-points, which is why it was used. *P*-values depicting the ranges at which the improvements to healthspan were statistically significant are graphed.

### Hazard ratio

We used the CoxPHFitter Python function for calculating hazard ratio. The CoxPHFitter() function in Python is part of the lifelines library and is used to fit a Cox Proportional Hazards model to survival data. This model estimates the hazard ratio for one or more covariates (predictors) with respect to the time until an event occurs (e.g., death, failure, etc.).

### Volcano plot

Volcano plot was created using normalized antibody array values. To determine the significance of differences, comparisons were made using an unpaired two-tailed *t*-test.

### Enrichment analysis

Enrichment analysis was conducted using the KOBAS-i web platform [81]. Only significant terms (corrected *p*-value <0.05) were visualized, using Kyoto Encyclopedia of Genes and Genomes (KEGG) pathways through KOBAS-i.

### Venn diagram

The Venn diagram was generated from values from normalized antibody array data using InteractiVenn [82]. Proteins were selected from each of the groups (1) 7-day treated male mice (sedentary and exercised), (2) 7-day treated female mice (sedentary and exercised), (3) 4-month treated male mice, and (4) 4-month treated female mice, if their *p*-value was significant (*p* > 0.05) compared to their group and gender-matched vehicle controls. Textual changes were made with Illustrator.

## Supplementary Materials

Supplementary Figures

Supplementary Tables

Supplementary Data 1

## References

[1] Bartke A, Wright JC, Mattison JA, Ingram DK, Miller RA, Roth GS. Extending the lifespan of long-lived mice. Nature. 2001; 414:412. 10.1038/35106646 11719795

[2] Brown-Borg HM, Borg KE, Meliska CJ, Bartke A. Dwarf mice and the ageing process. Nature. 1996; 384:33. 10.1038/384033a0 8900272

[3] Coschigano KT, Clemmons D, Bellush LL, Kopchick JJ. Assessment of growth parameters and life span of GHR/BP gene-disrupted mice. Endocrinology. 2000; 141:2608–13. 10.1210/endo.141.7.7586 10875265

[4] Coschigano KT, Holland AN, Riders ME, List EO, Flyvbjerg A, Kopchick JJ. Deletion, but not antagonism, of the mouse growth hormone receptor results in severely decreased body weights, insulin, and insulin-like growth factor I levels and increased life span. Endocrinology. 2003; 144:3799–810. 10.1210/en.2003-0374 12933651

[5] Migliaccio E, Giorgio M, Mele S, Pelicci G, Reboldi P, Pandolfi PP, Lanfrancone L, Pelicci PG. The p66shc adaptor protein controls oxidative stress response and life span in mammals. Nature. 1999; 402:309–13. 10.1038/46311 10580504

[6] Bjedov I, Rallis C. The Target of Rapamycin Signalling Pathway in Ageing and Lifespan Regulation. Genes (Basel). 2020; 11:1043. 10.3390/genes11091043 32899412 PMC7565554

[7] Miller RA, Harrison DE, Astle CM, Fernandez E, Flurkey K, Han M, Javors MA, Li X, Nadon NL, Nelson JF, Pletcher S, Salmon AB, Sharp ZD, et al. Rapamycin-mediated lifespan increase in mice is dose and sex dependent and metabolically distinct from dietary restriction. Aging Cell. 2014; 13:468–77. 10.1111/acel.12194 24341993 PMC4032600

[8] Bitto A, Ito TK, Pineda VV, LeTexier NJ, Huang HZ, Sutlief E, Tung H, Vizzini N, Chen B, Smith K, Meza D, Yajima M, Beyer RP, et al. Transient rapamycin treatment can increase lifespan and healthspan in middle-aged mice. Elife. 2016; 5:e16351. 10.7554/eLife.16351 27549339 PMC4996648

[9] Wojciechowski D, Wiseman A. Long-Term Immunosuppression Management: Opportunities and Uncertainties. Clin J Am Soc Nephrol. 2021; 16:1264–71. 10.2215/CJN.15040920 33853841 PMC8455033

[10] Gkioni L, Nespital T, Baghdadi M, Monzó C, Bali J, Nassr T, Cremer AL, Beyer A, Deelen J, Backes H, Grönke S, Partridge L. The geroprotectors trametinib and rapamycin combine additively to extend mouse healthspan and lifespan. Nat Aging. 2025; 5:1249–65. 10.1038/s43587-025-00876-4 40437307 PMC12270913

[11] Garza-Lombó C, Schroder A, Reyes-Reyes EM, Franco R. mTOR/AMPK signaling in the brain: Cell metabolism, proteostasis and survival. Curr Opin Toxicol. 2018; 8:102–10. 10.1016/j.cotox.2018.05.002 30417160 PMC6223325

[12] Widjaja AA, Lim WW, Viswanathan S, Chothani S, Corden B, Dasan CM, Goh JWT, Lim R, Singh BK, Tan J, Pua CJ, Lim SY, Adami E, et al. Inhibition of IL-11 signalling extends mammalian healthspan and lifespan. Nature. 2024; 632:157–65. 10.1038/s41586-024-07701-9 39020175 PMC11291288

[13] Nandurkar HH, Robb L, Begley CG. The role of IL-II in hematopoiesis as revealed by a targeted mutation of its receptor. Stem Cells. 1998 (Suppl 2); 16:53–65. 10.1002/stem.5530160708 11012177

[14] Cork BA, Li TC, Warren MA, Laird SM. Interleukin-11 (IL-11) in human endometrium: expression throughout the menstrual cycle and the effects of cytokines on endometrial IL-11 production in vitro. J Reprod Immunol. 2001; 50:3–17. 10.1016/s0165-0378(00)00089-9 11254938

[15] Ren C, Chen Y, Han C, Fu D, Chen H. Plasma interleukin-11 (IL-11) levels have diagnostic and prognostic roles in patients with pancreatic cancer. Tumour Biol. 2014; 35:11467–72. 10.1007/s13277-014-2459-y 25123265

[16] Healey N. Senolytics target cellular senescence - but can they slow aging? Nat Med. 2024; 30:2698–9. 10.1038/d41591-024-00067-5 39223384

[17] Demaria M, Ohtani N, Youssef SA, Rodier F, Toussaint W, Mitchell JR, Laberge RM, Vijg J, Van Steeg H, Dollé ME, Hoeijmakers JH, de Bruin A, Hara E, Campisi J. An essential role for senescent cells in optimal wound healing through secretion of PDGF-AA. Dev Cell. 2014; 31:722–33. 10.1016/j.devcel.2014.11.012 25499914 PMC4349629

[18] Conboy IM, Conboy MJ, Wagers AJ, Girma ER, Weissman IL, Rando TA. Rejuvenation of aged progenitor cells by exposure to a young systemic environment. Nature. 2005; 433:760–4. 10.1038/nature03260 15716955

[19] Conboy MJ, Conboy IM, Rando TA. Heterochronic parabiosis: historical perspective and methodological considerations for studies of aging and longevity. Aging Cell. 2013; 12:525–30. 10.1111/acel.12065 23489470 PMC4072458

[20] Rebo J, Mehdipour M, Gathwala R, Causey K, Liu Y, Conboy MJ, Conboy IM. A single heterochronic blood exchange reveals rapid inhibition of multiple tissues by old blood. Nat Commun. 2016; 7:13363. 10.1038/ncomms13363 27874859 PMC5121415

[21] Kim D, Kiprov DD, Luellen C, Lieb M, Liu C, Watanabe E, Mei X, Cassaleto K, Kramer J, Conboy MJ, Conboy IM. Old plasma dilution reduces human biological age: a clinical study. Geroscience. 2022; 44:2701–20. 10.1007/s11357-022-00645-w 35999337 PMC9398900

[22] Mehdipour M, Skinner C, Wong N, Lieb M, Liu C, Etienne J, Kato C, Kiprov D, Conboy MJ, Conboy IM. Rejuvenation of three germ layers tissues by exchanging old blood plasma with saline-albumin. Aging (Albany NY). 2020; 12:8790–819. 10.18632/aging.103418 32474458 PMC7288913

[23] Winiarska A, Kwella N, Stompór T. Heparin-induced thrombocytopenia as a cause of prolonged low platelet count in a patient with thrombotic thrombocytopenic purpura treated with plasmapheresis. Acta Biochim Pol. 2017; 64:375–6. 10.18388/abp.2016_1428 28380082

[24] de Wit Y, Rethans A, van Mierlo G, Wouters D, Ten Brinke A, Bemelman FJ, Zeerleder S. Plasma Exchange Therapy Using Solvent Detergent-Treated Plasma: An Observational Pilot Study on Complement, Neutrophil and Endothelial Cell Activation in a Case Series of Patients Suffering from Atypical Hemolytic Uremic Syndrome. Transfus Med Hemother. 2022; 49:288–97. 10.1159/000522137 37969865 PMC10642533

[25] Mokrzycki MH, Kaplan AA. Therapeutic plasma exchange: complications and management. Am J Kidney Dis. 1994; 23:817–27. 10.1016/s0272-6386(12)80135-1 8203364

[26] Puig L, Mazzara R, Torras A, Castillo R. Adverse effects secondary to the treatment with plasma exchange. Int J Artif Organs. 1985; 8:155–8. 4030133

[27] Mehdipour M, Etienne J, Chen CC, Gathwala R, Rehman M, Kato C, Liu C, Liu Y, Zuo Y, Conboy MJ, Conboy IM. Rejuvenation of brain, liver and muscle by simultaneous pharmacological modulation of two signaling determinants, that change in opposite directions with age. Aging (Albany NY). 2019; 11:5628–45. 10.18632/aging.102148 31422380 PMC6710051

[28] Yousef H, Conboy MJ, Morgenthaler A, Schlesinger C, Bugaj L, Paliwal P, Greer C, Conboy IM, Schaffer D. Systemic attenuation of the TGF-β pathway by a single drug simultaneously rejuvenates hippocampal neurogenesis and myogenesis in the same old mammal. Oncotarget. 2015; 6:11959–78. 10.18632/oncotarget.3851 26003168 PMC4494916

[29] Larson C, Oronsky B, Carter CA, Oronsky A, Knox SJ, Sher D, Reid TR. TGF-beta: a master immune regulator. Expert Opin Ther Targets. 2020; 24:427–38. 10.1080/14728222.2020.1744568 32228232

[30] Tzavlaki K, Moustakas A. TGF-β Signaling. Biomolecules. 2020; 10:487. 10.3390/biom10030487 32210029 PMC7175140

[31] Zeki S. The neurobiology of love. FEBS Lett. 2007; 581:2575–9. 10.1016/j.febslet.2007.03.094 17531984

[32] Breuil V, Trojani MC, Ez-Zoubir A. Oxytocin and Bone: Review and Perspectives. Int J Mol Sci. 2021; 22:8551. 10.3390/ijms22168551 34445256 PMC8395200

[33] Elabd C, Cousin W, Upadhyayula P, Chen RY, Chooljian MS, Li J, Kung S, Jiang KP, Conboy IM. Oxytocin is an age-specific circulating hormone that is necessary for muscle maintenance and regeneration. Nat Commun. 2014; 5:4082. 10.1038/ncomms5082 24915299 PMC4512838

[34] Jurek B, Neumann ID. The Oxytocin Receptor: From Intracellular Signaling to Behavior. Physiol Rev. 2018; 98:1805–908. 10.1152/physrev.00031.2017 29897293

[35] Yuan R, Meng Q, Nautiyal J, Flurkey K, Tsaih SW, Krier R, Parker MG, Harrison DE, Paigen B. Genetic coregulation of age of female sexual maturation and lifespan through circulating IGF1 among inbred mouse strains. Proc Natl Acad Sci U S A. 2012; 109:8224–9. 10.1073/pnas.1121113109 22566614 PMC3361401

[36] Whitehead JC, Hildebrand BA, Sun M, Rockwood MR, Rose RA, Rockwood K, Howlett SE. A clinical frailty index in aging mice: comparisons with frailty index data in humans. J Gerontol A Biol Sci Med Sci. 2014; 69:621–32. 10.1093/gerona/glt136 24051346 PMC4022099

[37] Dougherty JP, Springer DA, Gershengorn MC. The Treadmill Fatigue Test: A Simple, High-throughput Assay of Fatigue-like Behavior for the Mouse. J Vis Exp. 2016; 54052. 10.3791/54052 27286034 PMC4927751

[38] Klein SM, Vykoukal J, Lechler P, Zeitler K, Gehmert S, Schreml S, Alt E, Bogdahn U, Prantl L. Noninvasive in vivo assessment of muscle impairment in the mdx mouse model--a comparison of two common wire hanging methods with two different results. J Neurosci Methods. 2012; 203:292–7. 10.1016/j.jneumeth.2011.10.001 22015600

[39] Antunes M, Biala G. The novel object recognition memory: neurobiology, test procedure, and its modifications. Cogn Process. 2012; 13:93–110. 10.1007/s10339-011-0430-z 22160349 PMC3332351

[40] Cohen SJ, Stackman RW Jr. Assessing rodent hippocampal involvement in the novel object recognition task. A review. Behav Brain Res. 2015; 285:105–17. 10.1016/j.bbr.2014.08.002 25169255 PMC7008635

[41] Liu Y, Conboy MJ, Mehdipour M, Liu Y, Tran TP, Blotnick A, Rajan P, Santos TC, Conboy IM. Application of bio-orthogonal proteome labeling to cell transplantation and heterochronic parabiosis. Nat Commun. 2017; 8:643. 10.1038/s41467-017-00698-y 28935952 PMC5608760

[42] Liu C, Wong N, Watanabe E, Hou W, Biral L, DeCastro J, Mehdipour M, Aran K, Conboy MJ, Conboy IM. Mechanisms and Minimization of False Discovery of Metabolic Bioorthogonal Noncanonical Amino Acid Proteomics. Rejuvenation Res. 2022; 25:95–109. 10.1089/rej.2022.0019 35323026 PMC9063144

[43] Nikopoulou C, Parekh S, Tessarz P. Ageing and sources of transcriptional heterogeneity. Biol Chem. 2019; 400:867–78. 10.1515/hsz-2018-0449 30951493

[44] Nordstokke DW, Zumbo BD. A Cautionary Tale about Levene’s Tests for Equal Variances. J Educ Res Policy Stud. 2007; 7:1–14. https://files.eric.ed.gov/fulltext/EJ809430.pdf.

[45] Anisimov VN, Popovich IG, Zabezhinski MA, Egormin PA, Yurova MN, Semenchenko AV, Tyndyk ML, Panchenko AV, Trashkov AP, Vasiliev AG, Khaitsev NV. Sex differences in aging, life span and spontaneous tumorigenesis in 129/Sv mice neonatally exposed to metformin. Cell Cycle. 2015; 14:46–55. 10.4161/15384101.2014.973308 25483062 PMC4353070

[46] Harrison DE, Strong R, Allison DB, Ames BN, Astle CM, Atamna H, Fernandez E, Flurkey K, Javors MA, Nadon NL, Nelson JF, Pletcher S, Simpkins JW, et al. Acarbose, 17-α-estradiol, and nordihydroguaiaretic acid extend mouse lifespan preferentially in males. Aging Cell. 2014; 13:273–82. 10.1111/acel.12170 24245565 PMC3954939

[47] Strong R, Miller RA, Astle CM, Floyd RA, Flurkey K, Hensley KL, Javors MA, Leeuwenburgh C, Nelson JF, Ongini E, Nadon NL, Warner HR, Harrison DE. Nordihydroguaiaretic acid and aspirin increase lifespan of genetically heterogeneous male mice. Aging Cell. 2008; 7:641–50. 10.1111/j.1474-9726.2008.00414.x 18631321 PMC2695675

[48] Strong R, Miller RA, Antebi A, Astle CM, Bogue M, Denzel MS, Fernandez E, Flurkey K, Hamilton KL, Lamming DW, Javors MA, de Magalhães JP, Martinez PA, et al. Longer lifespan in male mice treated with a weakly estrogenic agonist, an antioxidant, an α-glucosidase inhibitor or a Nrf2-inducer. Aging Cell. 2016; 15:872–84. 10.1111/acel.12496 27312235 PMC5013015

[49] Habermehl TL, Underwood KB, Welch KD, Gawrys SP, Parkinson KC, Schneider A, Masternak MM, Mason JB. Aging-associated changes in motor function are ovarian somatic tissue-dependent, but germ cell and estradiol independent in post-reproductive female mice exposed to young ovarian tissue. Geroscience. 2022; 44:2157–69. 10.1007/s11357-022-00549-9 35349034 PMC8962938

[50] Nisbett KE, Gonzalez LA, Teruel M, Carter CS, Vendruscolo LF, Ragozzino ME, Koob GF. Sex and hormonal status influence the anxiolytic-like effect of oxytocin in mice. Neurobiol Stress. 2023; 26:100567. 10.1016/j.ynstr.2023.100567 37706061 PMC10495655

[51] Chu YL, Xu YR, Yang WX, Sun Y. The role of FSH and TGF-β superfamily in follicle atresia. Aging (Albany NY). 2018; 10:305–21. 10.18632/aging.101391 29500332 PMC5892684

[52] Ilnytska O, Argyropoulos G. The role of the Agouti-Related Protein in energy balance regulation. Cell Mol Life Sci. 2008; 65:2721–31. 10.1007/s00018-008-8104-4 18470724 PMC2748318

[53] Biddinger JE, Elson AET, Fathi PA, Sweet SR, Nishimori K, Ayala JE, Simerly RB. AgRP neurons mediate activity-dependent development of oxytocin connectivity and autonomic regulation. Proc Natl Acad Sci U S A. 2024; 121:e2403810121. 10.1073/pnas.2403810121 39585985 PMC11626166

[54] Carson DS, Guastella AJ, Taylor ER, McGregor IS. A brief history of oxytocin and its role in modulating psychostimulant effects. J Psychopharmacol. 2013; 27:231–47. 10.1177/0269881112473788 23348754

[55] Malek E, Rana PS, Swamydas M, Daunov M, Miyagi M, Murphy E, Ignatz-Hoover JJ, Metheny L, Kim SJ, Driscoll JJ. Vactosertib, a novel TGF-β1 type I receptor kinase inhibitor, improves T-cell fitness: a single-arm, phase 1b trial in relapsed/refractory multiple myeloma. Res Sq. 2023. 10.21203/rs.3.rs-3112163/v1 PMC11350185 39191755

[56] Lee HJ. Recent Advances in the Development of TGF-β Signaling Inhibitors for Anticancer Therapy. J Cancer Prev. 2020; 25:213–22. 10.15430/JCP.2020.25.4.213 33409254 PMC7783242

[57] Swindell WR. Meta-Analysis of 29 Experiments Evaluating the Effects of Rapamycin on Life Span in the Laboratory Mouse. J Gerontol A Biol Sci Med Sci. 2017; 72:1024–32. 10.1093/gerona/glw153 27519886

[58] Smith DL Jr, Elam CF Jr, Mattison JA, Lane MA, Roth GS, Ingram DK, Allison DB. Metformin supplementation and life span in Fischer-344 rats. J Gerontol A Biol Sci Med Sci. 2010; 65:468–74. 10.1093/gerona/glq033 20304770 PMC2854888

[59] Mohammed I, Hollenberg MD, Ding H, Triggle CR. A Critical Review of the Evidence That Metformin Is a Putative Anti-Aging Drug That Enhances Healthspan and Extends Lifespan. Front Endocrinol (Lausanne). 2021; 12:718942. 10.3389/fendo.2021.718942 34421827 PMC8374068

[60] Roberts MN, Wallace MA, Tomilov AA, Zhou Z, Marcotte GR, Tran D, Perez G, Gutierrez-Casado E, Koike S, Knotts TA, Imai DM, Griffey SM, Kim K, et al. A Ketogenic Diet Extends Longevity and Healthspan in Adult Mice. Cell Metab. 2017; 26:539–46.e5. 10.1016/j.cmet.2017.08.005 28877457 PMC5609489

[61] Bárcena C, Valdés-Mas R, Mayoral P, Garabaya C, Durand S, Rodríguez F, Fernández-García MT, Salazar N, Nogacka AM, Garatachea N, Bossut N, Aprahamian F, Lucia A, et al. Healthspan and lifespan extension by fecal microbiota transplantation into progeroid mice. Nat Med. 2019; 25:1234–42. 10.1038/s41591-019-0504-5 31332389

[62] Deng Z, Fan T, Xiao C, Tian H, Zheng Y, Li C, He J. TGF-β signaling in health, disease, and therapeutics. Signal Transduct Target Ther. 2024; 9:61. 10.1038/s41392-024-01764-w 38514615 PMC10958066

[63] Guo X, Wang XF. Signaling cross-talk between TGF-beta/BMP and other pathways. Cell Res. 2009; 19:71–88. 10.1038/cr.2008.302 19002158 PMC3606489

[64] Baba AB, Rah B, Bhat GR, Mushtaq I, Parveen S, Hassan R, Hameed Zargar M, Afroze D. Transforming Growth Factor-Beta (TGF-β) Signaling in Cancer-A Betrayal Within. Front Pharmacol. 2022; 13:791272. 10.3389/fphar.2022.791272 35295334 PMC8918694

[65] Davizon-Castillo P, McMahon B, Aguila S, Bark D, Ashworth K, Allawzi A, Campbell RA, Montenont E, Nemkov T, D'Alessandro A, Clendenen N, Shih L, Sanders NA, et al. TNF-α-driven inflammation and mitochondrial dysfunction define the platelet hyperreactivity of aging. Blood. 2019; 134:727–40. 10.1182/blood.2019000200 31311815 PMC6716075

[66] Dirks AJ, Leeuwenburgh C. Tumor necrosis factor alpha signaling in skeletal muscle: effects of age and caloric restriction. J Nutr Biochem. 2006; 17:501–8. 10.1016/j.jnutbio.2005.11.002 16517142

[67] Phillips T, Leeuwenburgh C. Muscle fiber specific apoptosis and TNF-alpha signaling in sarcopenia are attenuated by life-long calorie restriction. FASEB J. 2005; 19:668–70. 10.1096/fj.04-2870fje 15665035

[68] Chen M, Xiao L, Dai G, Lu P, Zhang Y, Li Y, Ni M, Rui Y. Inhibition of JAK-STAT Signaling Pathway Alleviates Age-Related Phenotypes in Tendon Stem/Progenitor Cells. Front Cell Dev Biol. 2021; 9:650250. 10.3389/fcell.2021.650250 33855026 PMC8039155

[69] Hu X, Li J, Fu M, Zhao X, Wang W. The JAK/STAT signaling pathway: from bench to clinic. Signal Transduct Target Ther. 2021; 6:402. 10.1038/s41392-021-00791-1 34824210 PMC8617206

[70] Fischer R, Kontermann RE, Pfizenmaier K. Selective Targeting of TNF Receptors as a Novel Therapeutic Approach. Front Cell Dev Biol. 2020; 8:401. 10.3389/fcell.2020.00401 32528961 PMC7264106

[71] Xue C, Yao Q, Gu X, Shi Q, Yuan X, Chu Q, Bao Z, Lu J, Li L. Evolving cognition of the JAK-STAT signaling pathway: autoimmune disorders and cancer. Signal Transduct Target Ther. 2023; 8:204. 10.1038/s41392-023-01468-7 37208335 PMC10196327

[72] Lindahl GE, Chambers RC, Papakrivopoulou J, Dawson SJ, Jacobsen MC, Bishop JE, Laurent GJ. Activation of fibroblast procollagen alpha 1(I) transcription by mechanical strain is transforming growth factor-beta-dependent and involves increased binding of CCAAT-binding factor (CBF/NF-Y) at the proximal promoter. J Biol Chem. 2002; 277:6153–61. 10.1074/jbc.M108966200 11748224

[73] Heinemeier K, Langberg H, Olesen JL, Kjaer M. Role of TGF-beta1 in relation to exercise-induced type I collagen synthesis in human tendinous tissue. J Appl Physiol (1985). 2003; 95:2390–7. 10.1152/japplphysiol.00403.2003 12923117

[74] Varga J, Jimenez SA. Modulation of collagen gene expression: its relation to fibrosis in systemic sclerosis and other disorders. Ann Intern Med. 1995; 122:60–2. 10.7326/0003-4819-122-1-199501010-00010 7985897

[75] Koyama Y, Xu J, Liu X, Brenner DA. New Developments on the Treatment of Liver Fibrosis. Dig Dis. 2016; 34:589–96. 10.1159/000445269 27332862 PMC4961096

[76] MacFarlane EG, Haupt J, Dietz HC, Shore EM. TGF-β Family Signaling in Connective Tissue and Skeletal Diseases. Cold Spring Harb Perspect Biol. 2017; 9:a022269. 10.1101/cshperspect.a022269 28246187 PMC5666637

[77] Smoktunowicz N, Alexander RE, Franklin L, Williams AE, Holman B, Mercer PF, Jarai G, Scotton CJ, Chambers RC. The anti-fibrotic effect of inhibition of TGFβ-ALK5 signalling in experimental pulmonary fibrosis in mice is attenuated in the presence of concurrent γ-herpesvirus infection. Dis Model Mech. 2015; 8:1129–39. 10.1242/dmm.019984 26138704 PMC4582104

[78] Tan Y, Mosallanejad K, Zhang Q, O'Brien S, Clements M, Perper S, Wilson S, Chaulagain S, Wang J, Abdalla M, Al-Saidi H, Butt D, Clabbers A, et al. IL11-mediated stromal cell activation may not be the master regulator of pro-fibrotic signaling downstream of TGFβ. Front Immunol. 2024; 15:1293883. 10.3389/fimmu.2024.1293883 38455057 PMC10917968

[79] Parasuraman S, Raveendran R, Kesavan R. Blood sample collection in small laboratory animals. J Pharmacol Pharmacother. 2010; 1:87–93. 10.4103/0976-500X.72350 21350616 PMC3043327

[80] McInnes L, Healy J, Melville J. UMAP: Uniform Manifold Approximation and Projection for Dimension Reduction. arXiv. 2020. 10.48550/arXiv.1802.03426

[81] Bu D, Luo H, Huo P, Wang Z, Zhang S, He Z, Wu Y, Zhao L, Liu J, Guo J, Fang S, Cao W, Yi L, et al. KOBAS-i: intelligent prioritization and exploratory visualization of biological functions for gene enrichment analysis. Nucleic Acids Res. 2021; 49:W317–25. 10.1093/nar/gkab447 34086934 PMC8265193

[82] Heberle H, Meirelles GV, da Silva FR, Telles GP, Minghim R. InteractiVenn: a web-based tool for the analysis of sets through Venn diagrams. BMC Bioinformatics. 2015; 16:169. 10.1186/s12859-015-0611-3 25994840 PMC4455604

