# Correction for: Sex-specific longitudinal reversal of aging in old frail mice

**DOI:** 10.18632/aging.206345

**Published:** 2025-11-30

**Authors:** Cameron Kato, Jessica Zheng, Cindy Quang, Sophia Siopack, Joana Cruz, Zachery R. Robinson, Nicole Fong, Zhixin A. Zhang, Patrick Young, Michael J. Conboy, Irina M. Conboy

**Affiliations:** 1 Department of Bioengineering and QB3 Institute, University of California, Berkeley, Berkeley, CA 94720, USA; 2 Current address: College of Medicine, University of the Philippines Manila, Manila, NCR 1000, Philippines

**Keywords:** lifespan, healthspan, Alk5 inhibitor, oxytocin, sex-specific differences

**This article has been corrected:** The authors would like to make an addition to the “Conflicts of Interest” section of the article.

