# Supplementary material for: Sex-specific longitudinal reversal of aging in old frail mice
Source: Aging (Albany NY). 2025 Aug 21;17(9):2252–77. doi: 10.18632/aging.206304 (PMC12517215; doi:10.18632/aging.206304)
Supplement: Supplementary Figures [file aging-17-9-206304-s001.pdf]

SUPPLEMENTARY FIGURES

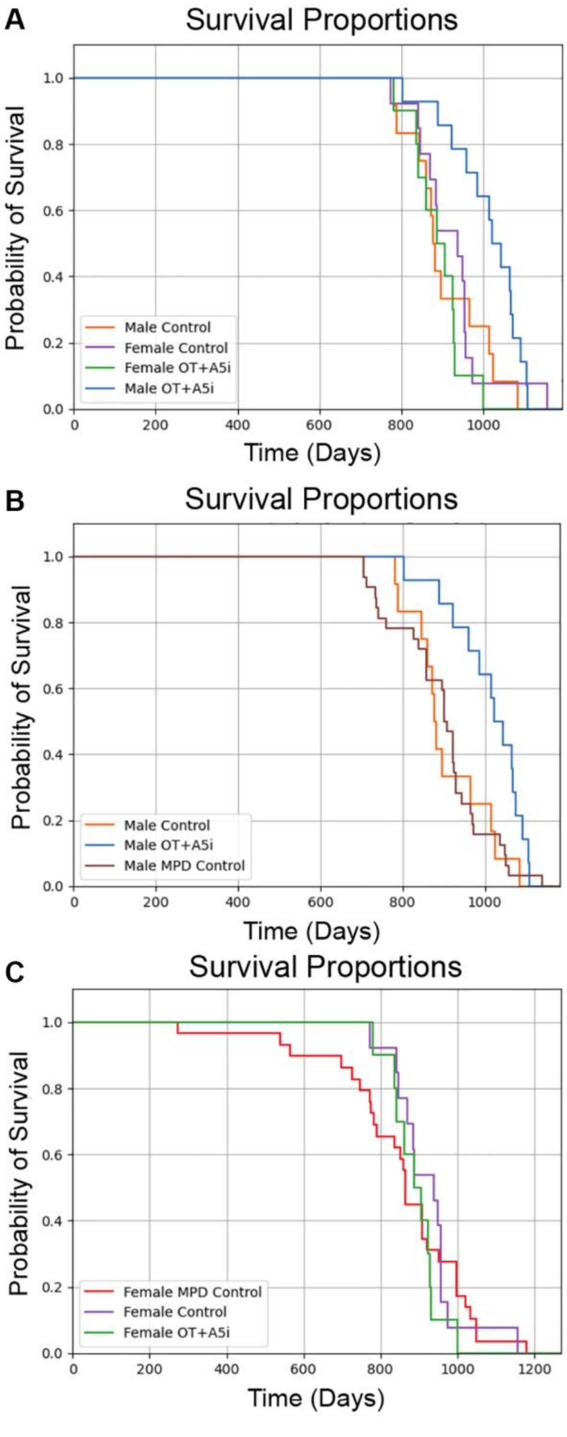

Supplementary Figure 1. Full length lifespan graphs. (**A**) Overlaid graphs of male and female lifespans. (**B**) Lifespans of male OT+A5i mice as compared to control mice graphs are plotted as Kaplan–Meier survival curves (p = 0.0109). Average lifespan of male OT+A5i: 1012 days. Average lifespan of male controls: 908 days. Hazard Ratio of full-length lifespan control vs. OT+A5i mice: 2.864. Control N = 12 and OT+A5i N = 14. MPD control includes published control data from the Mouse Phenome Database. (**C**) Lifespans of female OT+A5i as compared to control mice (p = 0.0109). Average lifespan of female OT+A5i: 890 days. Average lifespan of female controls: 923 days. Hazard Ratio of control to OT+A5i mice: 0.6109. Control N = 13 and OT+A5i N = 10.

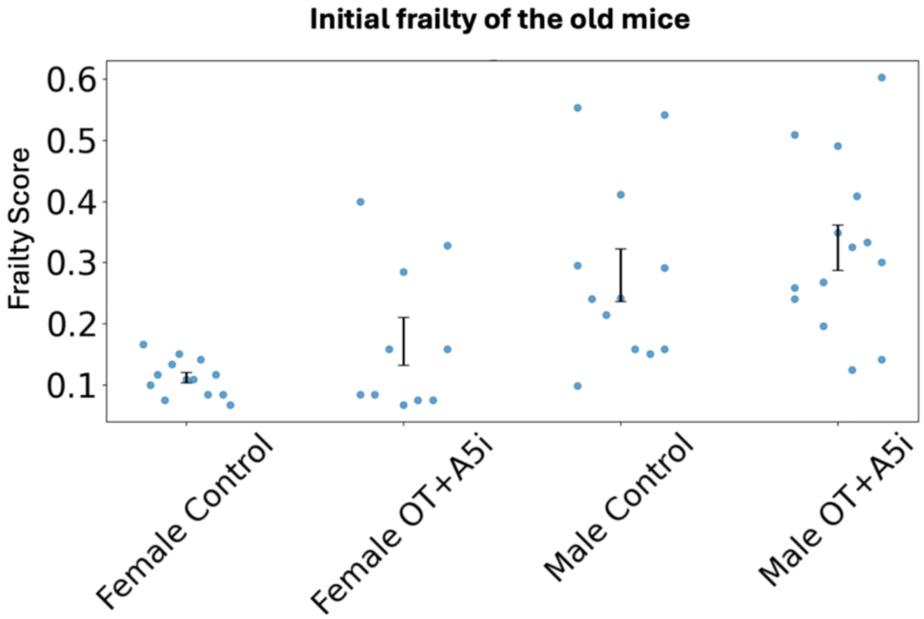

Supplementary Figure 2. Initial Frailty of old male and female mice. The initial frailties and standard errors from mean are shown for the 24–26-month-old mouse (each dot represents an animal) before administration of either OT+A5i or HBSS vehicle control. *p*-values were calculated via Welch's *T*-test. Male Control vs. Female Control: $p = 0.0026$. Male OT+A5i vs. Female OT+A5i: $p = 0.0093$. Male Control vs. Male OT+A5i: $p = 0.4340$. Female Control vs. Female OT+A5i: $p = 0.1634$. Male control mean variance: 0.280, 0.023. Female control mean variance: 0.112, 0.001. Male OT+A5i mean, variance: 0.325, 0.019. Female OT+A5i mean, variance: 0.171, 0.015.

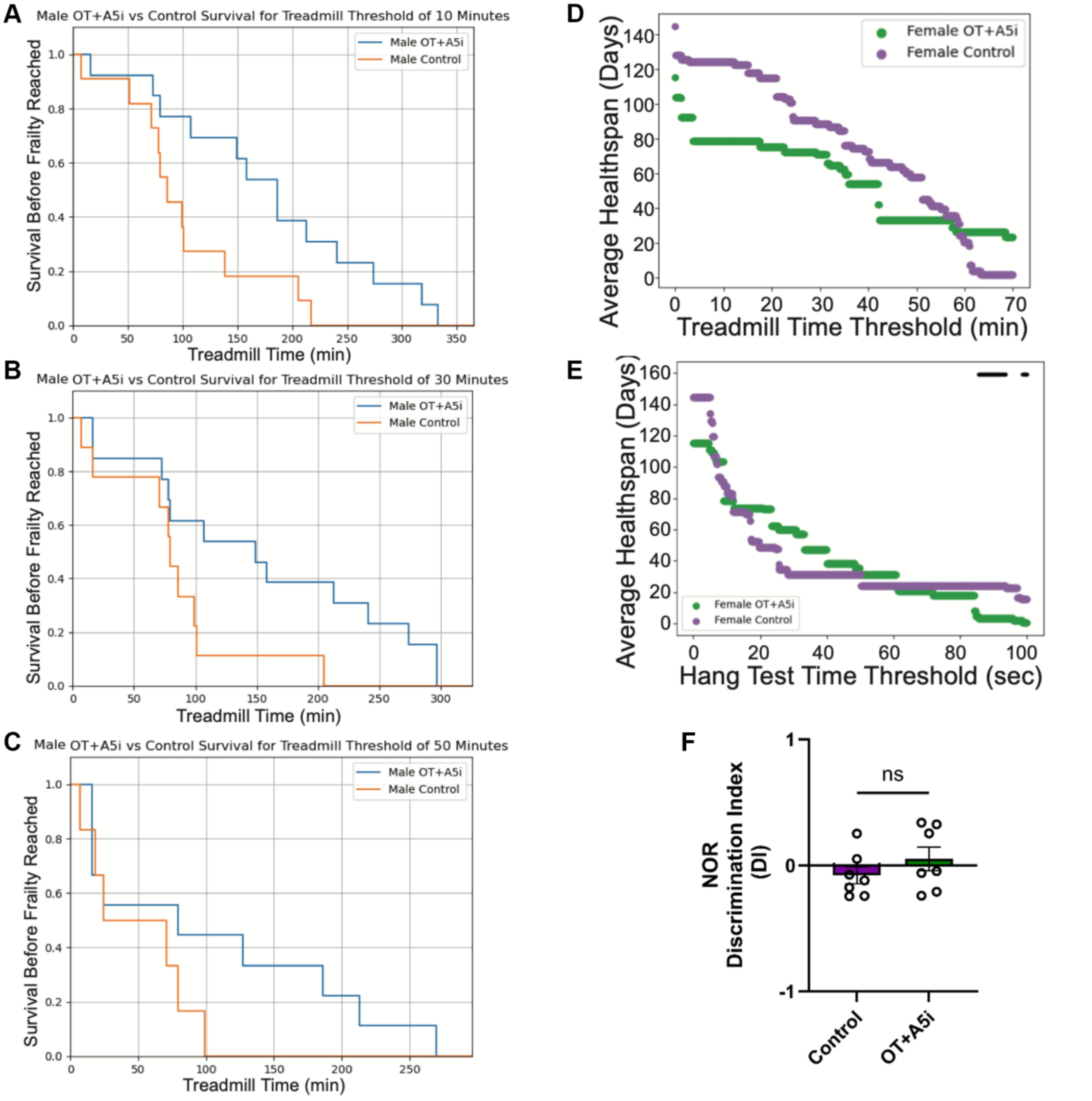

Supplementary Figure 3. Additional individual health parameters of old male and female mice. (**A**) Kaplan-Meier plot demonstrating the healthspan of the old male mice before they reach a healthspan threshold of a 10-minute running endurance. Average healthspan of controls = 102.9 days. Average healthspan of OT+A5i: 179.5 days. Control *N* = 12 and OT+A5i N = 14. (**p* = 0.0204). (**B**) Kaplan-Meier plot on the healthspan of old male mice before they reach a healthspan threshold of a 30-minute running endurance. Average healthspan of controls = 82.4 days. Average healthspan of OT+A5i: 153.7 days. Control *N* = 12 and OT+A5i *N* = 14. (**p* = 0.0311). (**C**) Kaplan-Meier plot demonstrating the healthspan of old male mice before they reach a healthspan threshold of a 50-minute running endurance. Average healthspan of controls = 49.7 days. Average healthspan of OT+A5i: 105.2 days. Control *N* = 12 and OT+A5i *N* = 14. (**p* = 0.1544: not statistically significant). (**D**) The average healthspans of OT+A5i and control old female mice across the range of treadmill running time thresholds from 0 to 70 minutes. Control *N* = 13 and OT+A5i *N* = 10. There were no thresholds at which a statistically significant difference was observed. (**E**) The average healthspans of OT+A5i and control old female mice across the range of hang test time thresholds from 0 to 100 seconds. Control *N* = 13 and OT+A5i *N* = 10. There were no thresholds at which a statistically significant difference was observed. (**F**) NOR test results for the old female mice at 4 months of OT/A5i. There was no statistical significance for novel object preference at 4-months of OT+A5i vs. HBSS administration. (Unpaired *t*-test *p* = 0.4139). OT/A5i *N* = 7, Control *N* = 7.

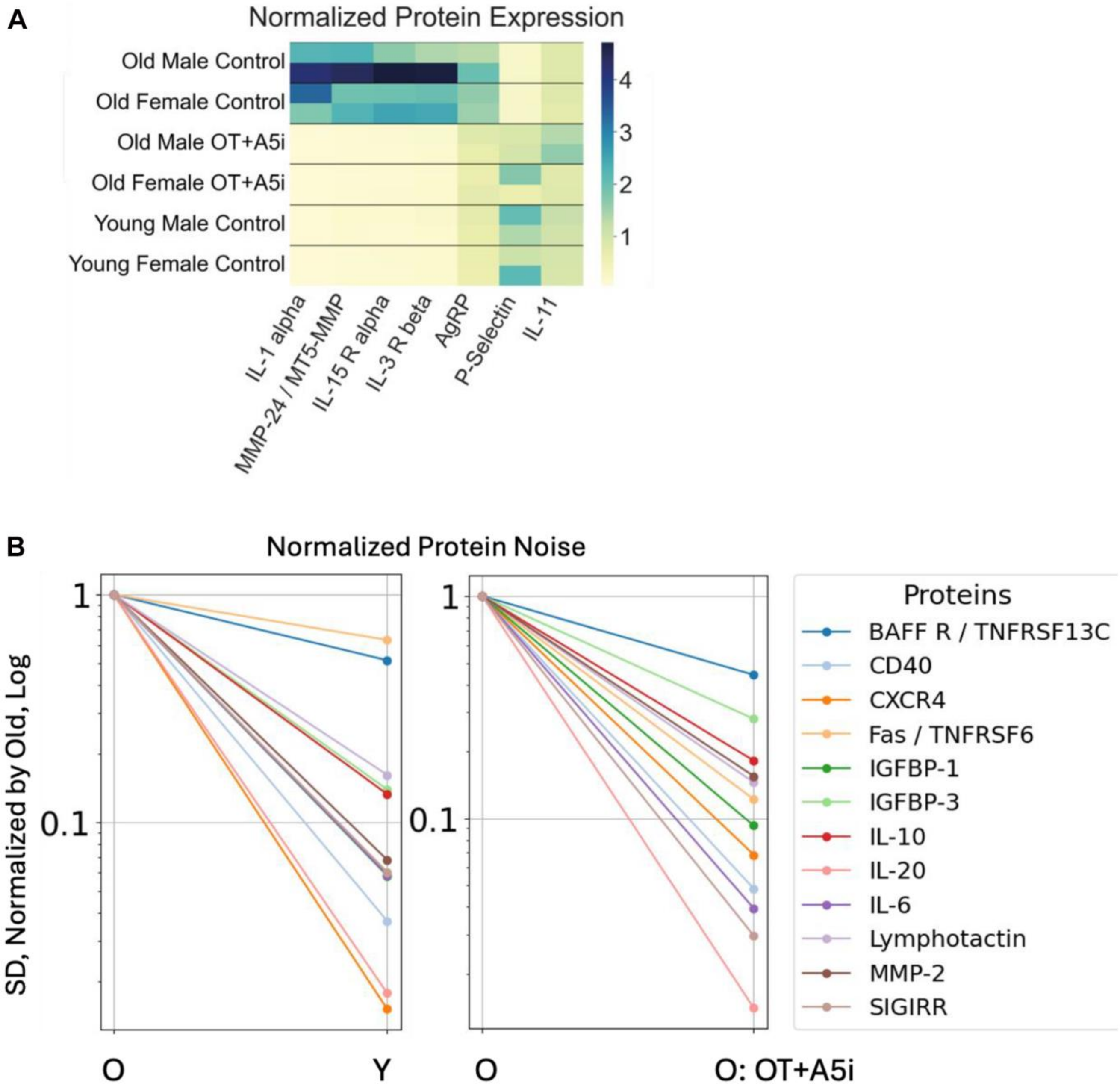

Supplementary Figure 4. Additional comparative proteomics analyses. (**A**) Heatmap of the designated by sex, differentially expressed *de-novo* synthesized proteins of control young, control old and OT+A5i treated old mice; IL-11 levels that are NS-not statistically different between the cohorts are also shown. (**B**) Combined for male and female mice comparison of the mean SDs of the proteins with lower noise in young and Old OT+A5i compared to old AHA-treated control groups, normalized by old control.

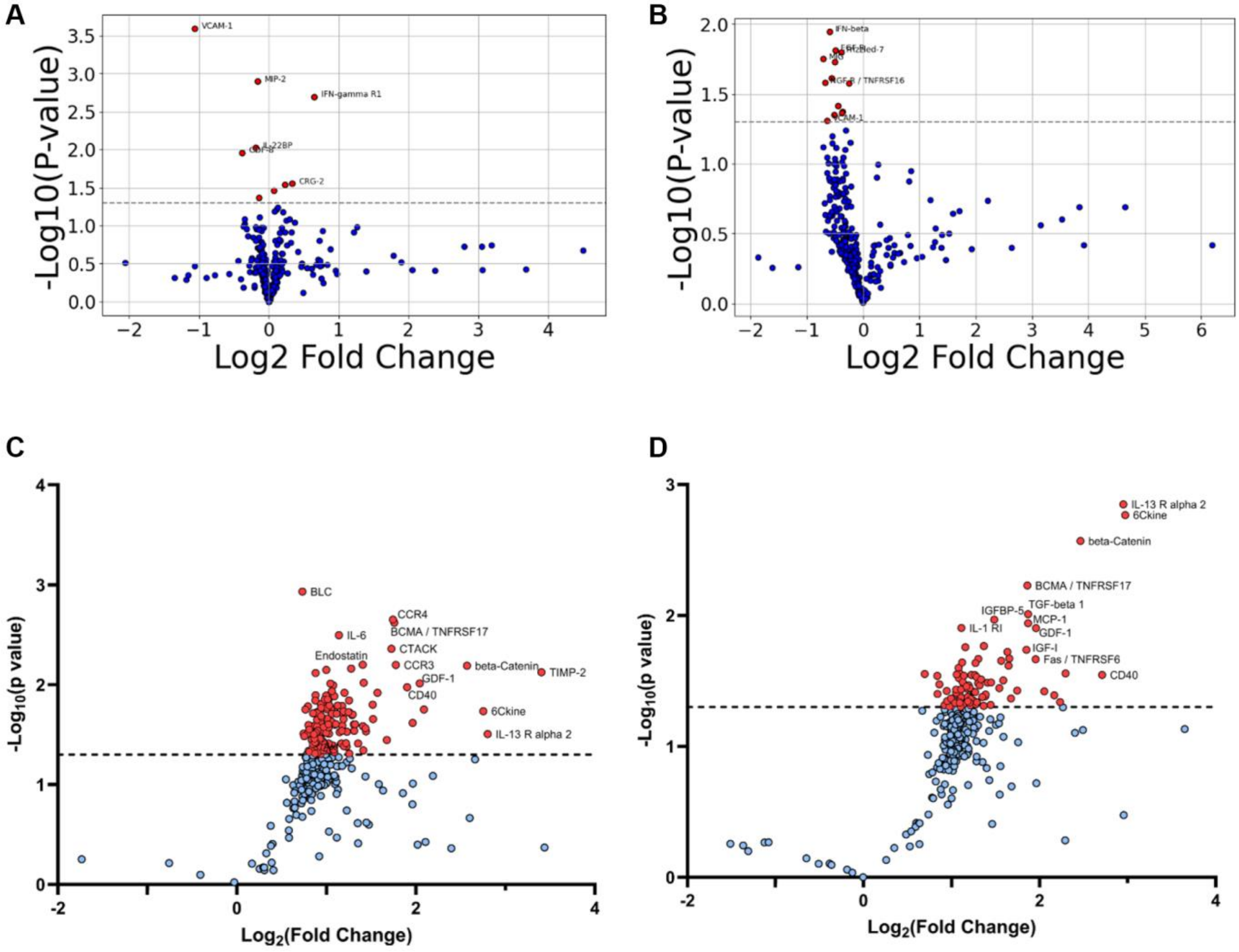

Supplementary Figure 5. Volcano plots on *de-novo* comparative BONCAT proteomics of sedentary old male and female mice at 7 days of OT+A5i or vehicle control. (**A**) Significant DEPs of female sedentary mice. (**B**) Significant DEPs of male sedentary mice. (**C**) Significant DEPs of female exercised mice. (**D**) Significant DEPs of male exercised mice. Red dots represent proteins with $p < 0.05$ OT+A5i vs. vehicle control; protein names and fold changes are shown. $n = 16$; 4 animals of each cohort: males control, females control, males OT+A5i, females OT+A5i.
