# Supplementary material for: Sex-specific longitudinal reversal of aging in old frail mice
Source: Aging (Albany NY). 2025 Aug 21;17(9):2252–77. doi: 10.18632/aging.206304 (PMC12517215; doi:10.18632/aging.206304)
Supplement: Supplementary Tables [file aging-17-9-206304-s002.pdf]

SUPPLEMENTARY TABLE

Supplementary Table 1. Effects of OT+A5i on fertility of 8–12-month-old mice.

Female age, months	Treatment	Number of females	Number of pups born	Number of pups weaned
8	HBSS	4	5	1
8	OT+A5i	5	12	5
10	HBSS	4	0	0
10	OT+A5i	5	9	8
12	HBSS	4	5	0
12	OT+A5i	5	7	3

Female mice of the indicted ages (8, 10, 12 months) were administered with either HBSS control vehicle or OT+A5i every day for 7 days. The female mice were bred with young (3–7 mo) male mice at 8, 10 and 12 months of age. The pups born and the pups successfully weaned per cohort are shown. Control N = 4 and OT+A5i N = 5.
